# Programmed cell death-index (PCDi) as a prognostic biomarker and predictor of drug sensitivity in cervical cancer: a machine learning-based analysis of mRNA signatures

**DOI:** 10.7150/jca.91798

**Published:** 2024-01-20

**Authors:** Wei Wang, Pengchen Chen, Songhua Yuan

**Affiliations:** 1 Department of Obstetrics and Gynecology, The First People's Hospital of Foshan, Foshan, 528000, Guangdong, China.; 2 Dongguan Maternal and Child Health Care Hospital, Postdoctoral Innovation Practice Base of Southern Medical University, Gongguan, 523125, Guangdong, China.

**Keywords:** programmed cell death, prognosis prediction, machine learning, nomogram, cervical cancer

## Abstract

**Purpose**: Cervical cancer is a significant public health concern, particularly in developing countries. Despite available treatment strategies, the prognosis for patients with locally advanced cervical cancer and beyond remains poor. Therefore, an accurate prediction model that can reliably forecast prognosis is essential in clinical setting. Programmed cell death (PCD) mechanisms are diverse and play a critical role in tumor growth, survival, and metastasis, making PCD a potential reliable prognostic marker for cervical cancer.

**Methods**: In this study, we created a novel prognostic indicator, programmed cell death-index (PCDi), based on a 10-fold cross-validation framework for comprehensive analysis of PCD-associated genes.

**Results**: Our PCDi-based prognostic model outperformed previously published signature models, stratifying cervical cancer patients into two distinct groups with significant differences in overall survival prognosis, tumor immune features, and drug sensitivity. Higher PCDi scores were associated with poorer prognosis. The nomogram survival model integrated PCDi and clinical characteristics, demonstrating higher prognostic prediction performance. Furthermore, our study investigated the immune features of cervical cancer patients and found that those with high PCDi scores had lower infiltrating immune cells, lower potential of T cell dysfunction, and higher potential of T cell exclusion. Patients with high PCDi scores were resistant to classic chemotherapy regimens, including cisplatin, docetaxel, and paclitaxel, but showed sensitivity to the inhibitor SB505124 and Trametinib.

**Conclusion**: Our findings suggest that PCD-related gene signature could serve as a useful biomarker to reliably predict prognosis and guide treatment decisions in cervical cancer.

## Introduction

Cervical cancer remains the most common gynecological cancer for women worldwide, with 604,000 incidences and 342,000 deaths reported by World Health Organization in its latest report 1. Unfortunately, developing regions such as Asia and Africa suffer a disproportionate burden of cervical cancer due to limited access to HPV vaccinations and early-cancer screening programs 2. While surgery is the primary treatment option for cervical cancer, patients diagnosed with locally advanced cervical cancer (LACC) with risk factors usually require adjuvant concurrent chemoradiotherapy (CCRT) after surgery. However, the heterogeneity of cervical cancer often results in variable clinical outcomes among patients 3.

Recent advancements in bioinformatics have led to the development of numerous prognostic gene signatures for cervical cancer 4-6. Nonetheless, the application of these prognostic signatures in clinical practice has been hindered by their poor accuracy and unreliability. Therefore, there is an urgent need for a high-quality molecular prognosis prediction model to aid in clinical treatment decision-making.

Programmed cell death (PCD) is a crucial biological process that regulates cell suicide through specific signaling cascades, unlike accident cell death (ACD) caused by external injury or unintentional damage 7, 8. Various types of PCD mechanisms have been identified, including apoptosis, lysosome-dependent cell death, pyroptosis, immunogenic cell death, necroptosis, ferroptosis, autophagy-dependent cell death, cuproptosis, anoikis, paraptosis, parthanatos, entotic cell death, netotic cell death, alkaliptosis, and oxeiptosis.

Apoptosis is a gene-regulated biological process that enables phagocytes to disassemble and digest injured cells without affecting surrounding cells 9-11. Paraptosis, mediated by mitogen-activated protein kinases (MAPKs) and inhibited by the multifunctional adaptor protein Alix, is induced by different natural compounds 12, 13. Lysosome-dependent cell death is triggered by hydrolytic enzymes like cathepsins released into cytosol for cellular component degradations following lysosomal membrane permeabilization 14-16. Pyroptosis, an inflammatory form of cell death, is activated by the inflammasome multiprotein complex, followed by the release of pro-inflammatory factors 17, 18. Netotic cell death is caused by the formation of neutrophil extracellular traps (NETs) which are composed of decondensed chromatin and bactericidal proteins 19. Immunogenic cell death results in an immune response characterized by the secretion of various types of damage-associated molecular patterns (DAMPs) caused by endoplasmic reticulum stress 20, 21. Necroptosis, another form of inflammatory cell death shares similar morphological features with necrosis but greatly depends on the signaling pathway involving RIPK3 and MLKL 22, 23. Entotic cell death is caused by a cell invading to an adjacent living cell, leading to the death of the inner cell 24. Parthanatos is a form of cell death that relies on the activation of poly(ADP-ribose)-polymerase (PARP) and the translocation of mitochondrial-associated apoptosis-inducing factor (AIF), leading to DNA fragmentation and chromatin condensation 25. Anoikis is a regulated cell death form triggered by the detachment of anchorage-dependent cells from the surrounding extracellular matrix (ECM) due to the loss of cell-matrix interaction 26. Ferroptosis is initiated by the accumulation of lipid peroxides balanced by the production of reaction oxygen species (ROS) and antioxidant system in an iron-dependent manner 27-29. Autophagy-dependent cell death is regulated by over 40 autophagy-related genes, driven by autophagic machinery 30. Alkaliptosis, a pH-dependent cell death form, is induced by intracellular alkalinization through NF-kappaB signaling pathway and carbonic anhydrase 9 (CA9) downregulation 31. Oxeiptosis is triggered by a high intracellular ROS level, leading to a caspase-independent cell death process through the regulation of KEAP1-PGAM5-AIFM1 pathway 32. Similar to ferroptosis, the accumulation of the heavy metal copper can induce mitochondrial stress, eventually leading to cell deaths eventually. This process has been termed cuproptosis 33, 34.

Recent advances in PCD studies have led to the development of novel anticancer strategies and drugs that promote cell death, showing promising results in cancer treatment. Specifically, targeting overexpressed proteins like BCL-2/BCL-XL and MCL1 enables the therapeutic induction of apoptosis in tumors 11. Additionally, a recent study has proposed a novel approach to overcome drug resistance in chemotherapy treatment by inducing ferroptosis 27. Small molecules targeting caspase-1 can trigger pyroptosis, leading to the destruction of tumor cells in colorectal cancer 35. Furthermore, sensitizing detached tumor cells to anoikis can prevent tumor metastasis, making it a potential strategy in cancer therapy 36, 37. Recent studies have reported promising findings have been reported in killing cervical cancer cells by activating PCD mechanisms. Evidence from *in vivo* experiments have shown the significant impact of Nrf2 on the metastasis of cervical cancer by the enhancement of epithelial-mesenchymal transition (EMT) and anoikis resistance 38. Moreover, the potential therapeutic agents have been proposed to actively induce PCD activities. It has been reported that necroptosis could be induced in cervical cancer cells by a small anti- cancer agent called RETRA (REactivation of Transcriptional Reporter Activity) 39. Besides, as a natural bioflavonoid found in many medicinal herbs/plants, Pinostrobin (PN) exerted anticancer effect to eliminate cervical cancer cells by ROS-dependent apoptosis 40.

Cell homeostasis in multicellular organisms is maintained through the intricate processes of diverse PCDs 8. However, PCD mechanisms are often impaired in cancer cells, which allows them to resist and evade various forms of gene-regulated cell death, leading to tumor growth, progression, and metastasis 41. Our study presents a comprehensive exploration of the intricate relationship between PCD and cervical cancer. By integrating survival-associated genes, we have established a high-quality molecular prognostic model based on a novel indicator known as the programmed cell death index (PCDi), which holds great promise for predicting prognosis and selecting therapeutic regimens for cervical cancer patients. Our study identifies the heterogeneity of cervical cancer based on different PCD mechanisms, contributing to a deeper understanding of the disease and holding significant clinical implications. The development of PCDi highlights the potential for personalized medicine in the management of cervical cancer, ultimately leading to improved patient outcomes.

## Materials and Methods

### Data collection

PCD processes are regulated by various PCD-related genes. To create an ultimate gene list, we curated regulatory genes for fifteen PCD mechanisms from review articles and databases. Genes related to apoptosis, lysosome-dependent cell death, netotic cell death, entotic cell death, parthanatos, autophagy, alkaliptosis, and oxeiptosis were extracted based on a curated gene list 42. Additionally, pyroptosis- and immunogenic cell death-related genes were extracted from prior reviews 43, 44. Gene related to paraptosis, anokikis, and necroptosis with a relevance score > 0.4 were downloaded from GeneCards database (https://www.genecards.org/) 45. Furthermore, ferroptosis- and cuproptosis-related genes were downloaded from FerrDb database (http://www.zhounan.org/ferrdb/current/), only driver and suppressor datasets were extracted 46. The ultimate gene list contains a total of 1,949 PCD-related genes, including 580 apoptosis-related genes, 508 anoikis-related genes, 483 ferroptosis-related genes, 11 cuproptosis-related genes, 209 necroptosis-related genes, 33 pyroptosis-related genes, 34 immunogenic cell death-related genes, 367 autophagy-related genes, 17 paraptosis-related genes, 9 parthanatos-related genes, 15 entotic cell death-related genes, 8 netotic cell death-related genes, 7 alkaliptosis-related genes, 5 oxeiptosis- related genes, and 220 lysosome-dependent cell death-related genes (Supplementary Table S1).

Four datasets from different research institutes were collected for the following analysis. Using Xena platform from USCS Genomics Institute (https://xenabrowser.net/datapages/), we obtained a combined cervical cancer cohort of The Cancer Genome Atlas (TCGA) and The Genotype-Tissue Expression (GTEx) samples 47. To ensure consistency and comparability of clinical data, cancer stage information of patients was standardized according to 2009 The International Federation of Gynecology and Obstetrics (FIGO) Staging Classification (Supplementary Fig. 1). Two tumor samples were excluded because they came from metastatic tumor tissues, and five tumor samples were excluded because of a lack of mRNA expression data (Supplementary Fig. 2A). Molecular and clinical data from CGCI- HTMCP-CC dataset were accessed through National Cancer Institute's Genome Data Commons (https://gdc.cancer.gov/about-data/publications/CGCI-HTMCP-CC-2020) 48. Tumor tissues of five patients were excluded because they were ultimately not diagnosed with cervical cancer (Supplementary Fig. 2B). Through Gene Expression Omnibus (GEO) database, microarray and clinical data from GSE52904 and GSE44001 were collected 49, 50. For high-dimensional data visualization, t-distributed stochastic neighbor embedding (t-SNE) method was employed, using the R package “*tsne*”.

### Identification of the differentially expressed PCD-related genes

To identify differentially expressed genes (DEGs), we utilized samples from TCGA- GTEx cervical cancer cohort, consisting of 303 tumor tissues and 13 normal tissues. Raw transcriptome count data was analyzed using the R packages “edgeR”, “limma”, and “DESeq2” 51-53. A gene identified by at least two algorithms with the selection criteria of adjusted p-value or FDR < 0.05 and |Log2FC| >= 2, was considered as a significant DEG. The absolute value of the logarithm base 2 of the fold change (FC) greater than or equal to 2 is a common criterion for identifying DEGs.

### Construction of the PCD-related gene signature by machine learning algorithms

Survival-associated DEGs were screened using univariate Cox regression with a significant threshold of p-value < 0.1. Currently, many types of machine learning were available with unique algorithm deign. Without systematic comparison, it was unfair to rely on a particular machine learning algorithm for the model building. We employed ten machine learning algorithms, including survival support vector machine (survivalSVM), elastic network (Enet), least absolute shrinkage and selection operator (LASSO), ridge regression (Ridge), random survival forest (RSF), stepwise Cox regression analysis (stepwiseCox), fitting Cox models by likelihood based boosting (CoxBoost), generalized boosted regression modeling (GBM), partial least squares regression for Cox (plsRcox), and supervised principal components (SuperPC) based on a 10-fold cross-validation framework to identify the optimal gene signature for cervical cancer (Supplementary Method). A total of 101 combination results were generated. We evaluated the performance of prognostic signatures using a Rank Score, which was calculated using the following formula:



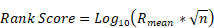



R_mean_ stands for the average rank and n denotes the number of genes in the signature model. Average area under the curve (AUC) of GSE52904 was the mean of 1-year and 2-year AUC values from time-dependent receiver operating characteristic (ROC) analysis. Average AUC of other datasets was the mean of 1-year, 2-year, 3-year, and 5-year AUC values from time-dependent ROC analysis. We chose the optimal signature model with lowest Rank Score, which had a reasonable number of genes and high average rank of Harrell's concordance index (C-index) and AUC value.

In this study, the prognostic gene signature was established through a combination of two machine learning algorithms stepwiseCox and Enet. Ten genes were identified by stepwiseCox algorithm with backward approach to yield the optimal model with minimum Akaike information criterion (AIC). Subsequently, the regression model was finalized using Enet algorithm with a parameter alpha set to 0.5. For each patient, a PCDi score was calculated using the following formula:



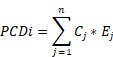



C_j_ stands for the coefficient and E_j_ denotes the expression value of each PCD-related gene. To facilitate cross-comparisons of PCDi across the datasets, a linear transformation was employed for data normalization. The normalized PCDi scores had a range from 0 to 1. Based on the median PCDi, cervical cancer patients were separated into PCDi-High and PCDi-Low groups. The R package “*stats*” was used to performance principal component (PCA) analysis for visualizing high-dimensional data. Kaplan-Meier (KM) analysis was used to investigate the survival difference between two compared groups. The R packages “*survival*” and “*survminer*” were implemented for survival analysis and result visualization.

### Functional annotation and enrichment analysis

The R package “*clusterProfiler*” was used to perform functional annotation analysis of DEGs based on Gene Ontology (GO) and Kyoto Encyclopedia of Genes and Genomes (KEGG) databases 54. REVIGO algorithm was implemented to remove redundant GO terms 55. Gene set enrichment analysis (GSEA) was performed to identify the significant enriched signaling pathways in association with PCDi subgroups.

### Establishment of the nomogram

The R package “*regplot*” was used to develop a nomogram survival model for cervical cancer that integrated PCDi and clinical features, including FIGO cancer stage and age at diagnosis. We evaluated the efficacy of the nomogram in prognosis prediction using the R packages “*rmda*” and “*rms*” for model calibration and decision curve analysis (DCA). The performance of the nomogram was also assessed by AUC value obtained through time-dependent ROC analysis with the R package “*timeROC*”.

### Tumor cell infiltration analysis and drug sensitivity prediction

Infiltrating immune cells in cervical cancer were estimated using TIMER2.0 platform 56. Tumor Immune Dysfunction and Exclusion (TIDE) algorithm was used to estimate the potential of tumor immune escape and response 57. The lists of gene signatures of 28 tumor infiltrating lymphocytes and genes encoding for immunomodulators and chemokines were downloaded from TISIDB database (http://cis.hku.hk/TISIDB/index.php) 58. Single-sample gene set enrichment analysis (ssGSEA) was used to calculate the enrichment scores of 28 tumor infiltrates. The R package “*oncoPredict*” was utilized to predict drug sensitivities for individual cervical cancer patients 59.

### Formalin-fixed and paraffin-embedded (FFPE) samples acquisition and immunohistochemistry (IHC) staining

Four FFPE samples were collected from patients diagnosed with cervical cancer from January 2023 to October 2023 at The First People's Hospital of Foshan. The samples of surgically resected cancerous and normal tissues were preserved after 4% formalin fixation and paraffin embedding treatment. Five µm thick sections were analyzed by IHC staining, as previously described 60. Briefly, heat-induced antigen retrieval was performed using 10 mM sodium citrate buffer, pH 6.5. Peroxidase activity was quenched with 3% H2O2 and tissues were blocked in 5% bovine serum albumin for 1 hour. Primary MMP1 (1:2000) (10371-2-AP, Proteintech) was added and sections were incubated overnight at 4 °C. Horseradish peroxidase-conjugated secondary antibody (1:1000) (K8002, Dako Corp., Ltd) and DAB were used for detection. Slides were counterstained with hematoxylin.

### Statistical analysis and data visualization

R software (http://www.R-project.org) was used for all other statistical analyses, such as Spearman correlation, Kruskal-wallis test, and Mann-Whitney test. Data visualization was carried out using the R package “*ggstatsplot*”, “*ggplot2*”, and Sangerbox platform (http://www.sangerbox.com/home.html).

## Results

### Landscape of PCD-related genes for cervical cancer

**Figure 1:**
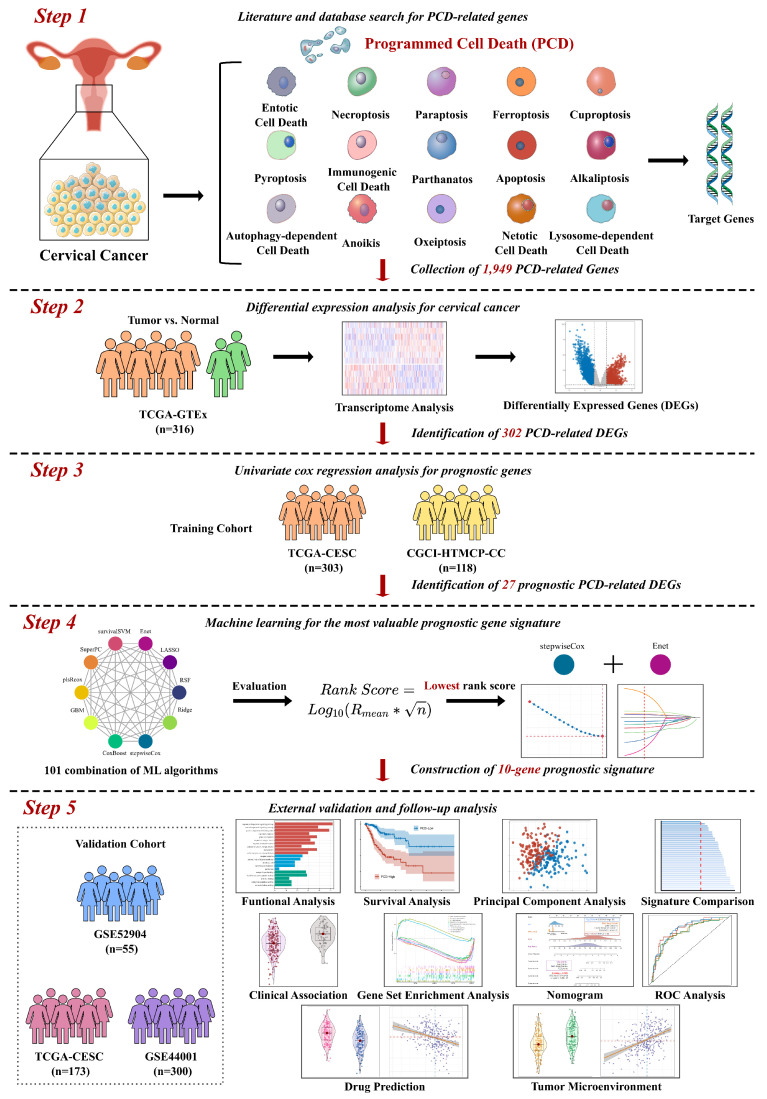
Computational workflow for developing the PCD-related prognostic signature for cervical cancer. The workflow includes five major steps. Firstly, PCD-related genes were identified through literature and database search. Secondly, DEGs were identified using TCGA-CESC cervical cancer dataset. Thirdly, univariate Cox regression was performed to identify prognostic genes. Fourthly, machine learning algorithms were used to identify the most valuable prognostic gene signature. Finally, independent validation and follow-up analysis were conducted.

In this study, we aimed to develop a reliable prognostic signature for cervical cancer based on the expression of PCD-related genes. Different PCD mechanisms regulate cell elimination that plays a crucial role in cancer development and therapy. PCD-related DEGs are genes that show significant changes in expression levels between cancer and normal tissues, and may reflect the PCD status of cancer cells. We hypothesized that PCD-related DEGs could serve as potential biomarkers for predicting the survival outcomes of cervical cancer patients. To test this hypothesis, we performed the following analyses. We collected 303 and 118 cervical cancer patients from TCGA- CESC and CGCI-HTMCP-CC datasets respectively, as the training cohort. We also collected 55, 173, and 300 patients from GSE52904, TCGA-CESC, and GSE44001 datasets respectively, as the validation cohort. We designed a computational workflow consisting of five major steps to develop the PCD-related prognostic signature for cervical cancer (Figure 1). In the first step, we compiled an ultimate gene list with 1,949 regulatory genes associated with fifteen PCD patterns based on literature and database search. In the second step, we identified PCD-related DEGs from the ultimate gene list by performing differential expression analysis of TCGA-GTEx dataset. In the third step, we further narrowed down the target genes by selecting prognosis-associated genes from the PCD-related DEGs using univariate Cox regression. In the fourth step, we established the most valuable PCD-related prognostic gene signature by applying the combination of 10 machine learning algorithms based on 10-fold cross validation framework. In the last step, we conducted a group of follow-up analyses to demonstrate the importance of PCD-related prognostic gene signature. More importantly, we used independent datasets to further validate the prognosis prediction of the gene signature.

**Figure 2:**
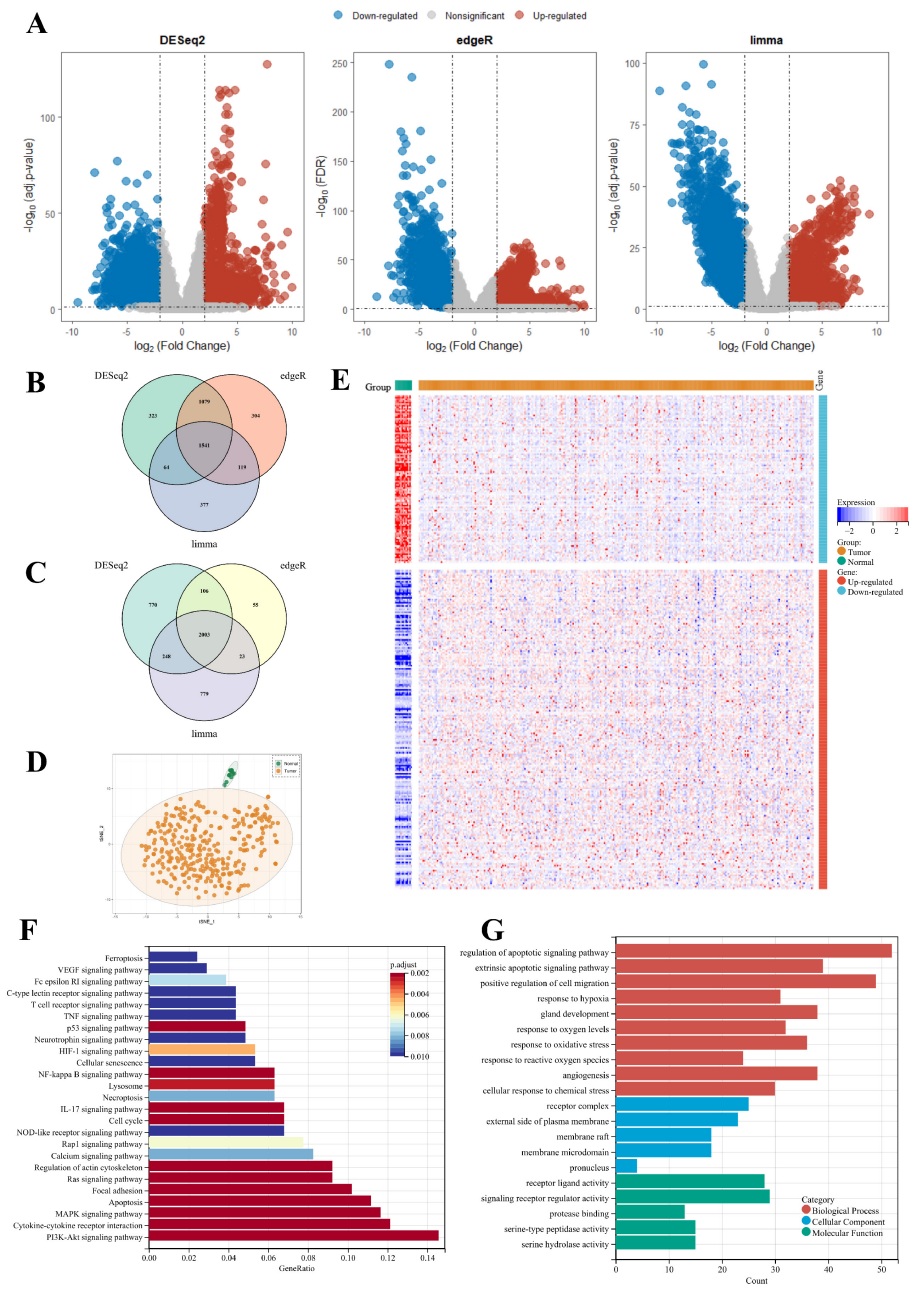
Comprehensive analysis of genes associated to fifteen types of PCD patterns for cervical cancer. (A) Volcano plots displaying the DEGs identified by algorithms DESeq2, edgeR, and limma algorithms. (B) Venn Diagram showing up-regulated DEGs in TCGA-CESC dataset. (C) Venn Diagram showing down-regulated DEGs in TCGA-CESC dataset. (D) t-SNE plot of transcriptomic data of 302 PCD-related DEGs in tumor and normal tissues. (E) Heatmap of PCD-related DEGs between tumor and normal tissues. (F) KEGG pathway annotation analysis based on PCD-related DEGs. (G) GO term annotation analysis based on PCD-related DEGs.

Using three different algorithms with selection criteria of adjusted p-value or FDR < 0.05 and |Log2FC| >= 2, we identified 2,803 up-regulated genes and 2,380 down-regulated genes in TCGA-CESC cervical cancer dataset (Figure 2A to C, Supplementary Table S2). Among the DEGs, a total of 302 PCD-related genes were identified, including 198 up-regulated 104 down-regulated genes. A heatmap of Z-score transformed expression levels between tumor and normal tissues is showed in Figure 2E, and two groups are well-separated (Figure 2D). Notably, KEGG pathway analysis revealed that the DEGs were not only implicated in cell death processes such as ferroptosis and necroptosis, but also in the signaling pathways like PI3K-Akt and NF-kappaB pathways, which play a critical role in regulating cell growth and survival (Figure 2F). Furthermore, GO functional annotation analysis showed that the DEGs are involved in the biological processes relating to oxygen level and ROS, which are pivotal mediators of multiple PCD mechanisms (Figure 2G).

### PCD-related prognostic gene signature

**Figure 3:**
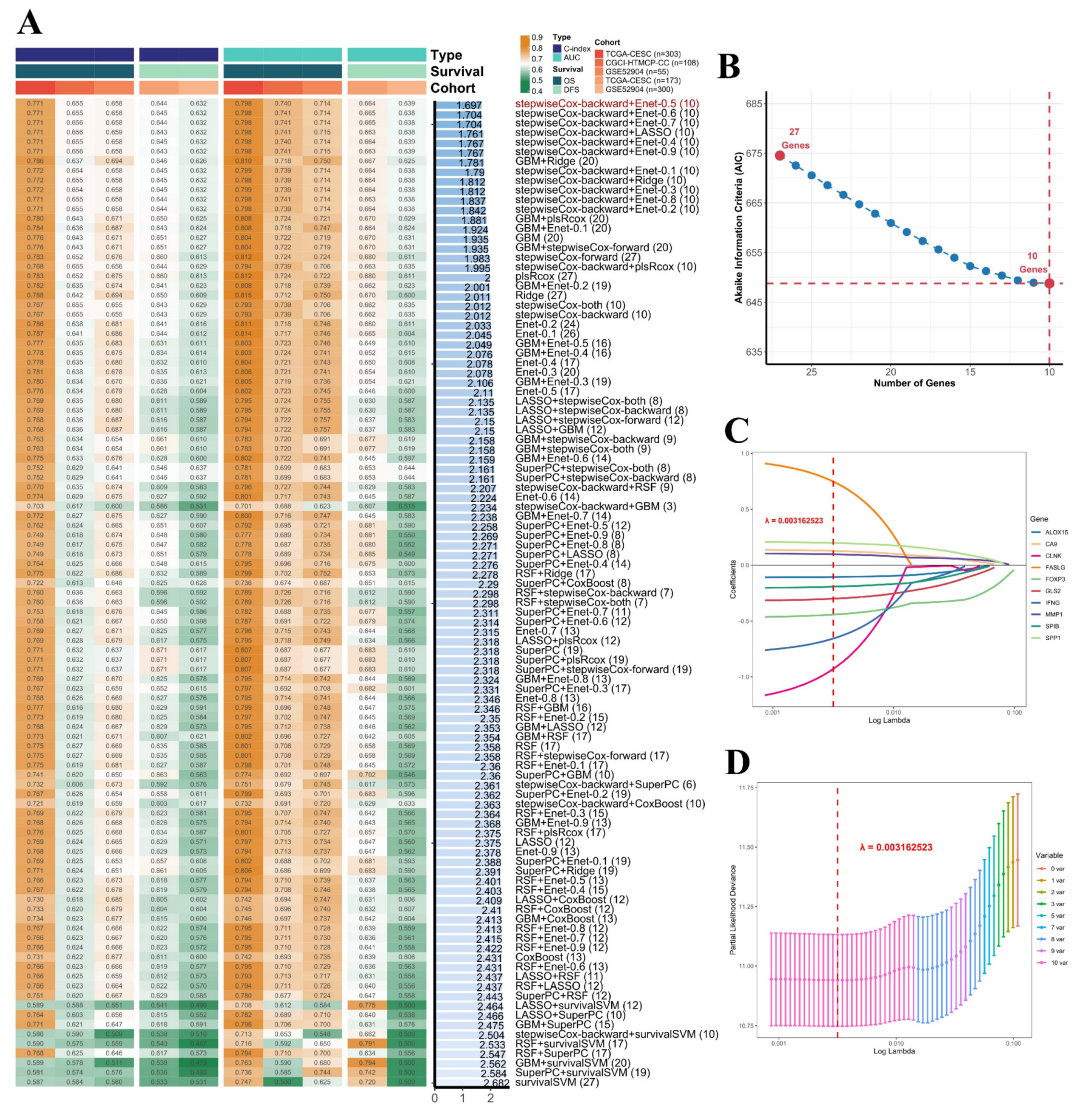
Development of the PCD-related prognostic signature by machine learning algorithms. (A) A total of 101 combinations of machine learning algorithms were used to identify the most valuable prognostic signature for cervical cancer. The C-index and AUC values of each dataset were displayed, and the Rank Score was calculated for each combination based on the rank average and the number of genes in the model. (B) The change of AIC value in stepwiseCox algorithm with a backward approach. (C) The selection of 10 PCD-related genes using Enet algorithm. (D) The selection of lambda.min value to minimize cross-validation error in regression analysis.

The overall survival (OS) data from two datasets within the training cohort were analyzed using univariate Cox regression analysis to identify survival-associated genes among 302 PCD-related DEGs. Applying a significant threshold of p-value < 0.1, 80 and 62 genes were identified in TCGA-CESC and CGCI-HTMCP-CC datasets respectively. Twenty-seven genes were consistently identified in both datasets and were subsequently utilized to construct the most robust prognostic gene signature (Supplementary Fig. S3). This procedure was achieved through the combinations of 10 machine learning algorithms, based on 10-fold cross-validation framework. The finalized PCD-related prognostic signature with best performance was established by the combination of stepwiseCox and Enet algorithms (Figure 3, Supplementary Table S3).

The PCD-related prognostic signature consisted of 10 genes that were up-regulated in tumor tissues (Supplementary Fig. S4). Among these genes, three genes were related to anoikis (SPP1, SPIB, FASLG), one gene was related to netotic cell death-related (MMP1), four genes were related to ferroptosis (ALOX15, GLS2, CA9, IFNG), two genes were related to apoptosis (IFNG, FASLG), two genes were related to immunogenic cell death (FOXP3, IFNG), one gene was related to necroptosis (FASLG), one gene was related to lysosome-dependent cell death (CLNK), one gene was related to alkaliptosis (CA9 ), and one gene related to autophagy-dependent cell death (IFNG). Notably, FALSG, CA9, and IFNG were involved in multiple PCD mechanisms.

We performed KM analysis to assess the association between the expression of genes in the PCD-related prognostic signature and OS. Using a median cutoff, we founded that all genes, except for FASLG, IFNG and ALOX15, had significant impact on OS (Log-rank test, p < 0.1, Supplementary Fig. S5). A novel prognostic indicator, called PCDi was derived from the 10-gene signature model. The PCDi score for each patient was calculated as the sum of the expression levels of 10 genes, weighted by the coefficients derived from Enet regression model. The formula for the calculation of PCDi is shown below. PCDi = (0.201458 * SPP1 exp.) + (0.098878 * MMP1 exp.) + (0.130493 * CA9 exp.) + (-0.108410 * ALOX15 exp.) + (0.304840 * GLS2 exp.) + (0.449150 * FOXP3 exp.) + (0.198570 * SPIB exp.) + (0.781472 * FALSG exp.) + (0.675460 * IFNG exp.) + (0.988550 * CLNK exp.). Based on the median PCDi, we stratified the patients from the TCGA-CESC dataset into two subgroups, PCDi-High (n = 152) and PCDi-Low (n=151). Statistical analysis was performed to investigate the associations between PCDi and clinical characteristics in cervical cancer patients (Supplementary Fig. S6A). Out analysis revealed that PCDi was significantly associated with FIGO clinical stage (Supplementary Fig. S6B), tumor size (Supplementary Fig. S6C), tumor metastasis (Supplementary Fig. S6E), and survival status (Supplementary Fig. S6F and G). Notably, even within the same clinical stage, patients from different PCDi subgroup have shown significant OS differences (Supplementary Fig. S7 and S8). However, PCDi was not associated with lymph node invasion in cervical cancer (Supplementary Fig. S6D).

**Figure 4:**
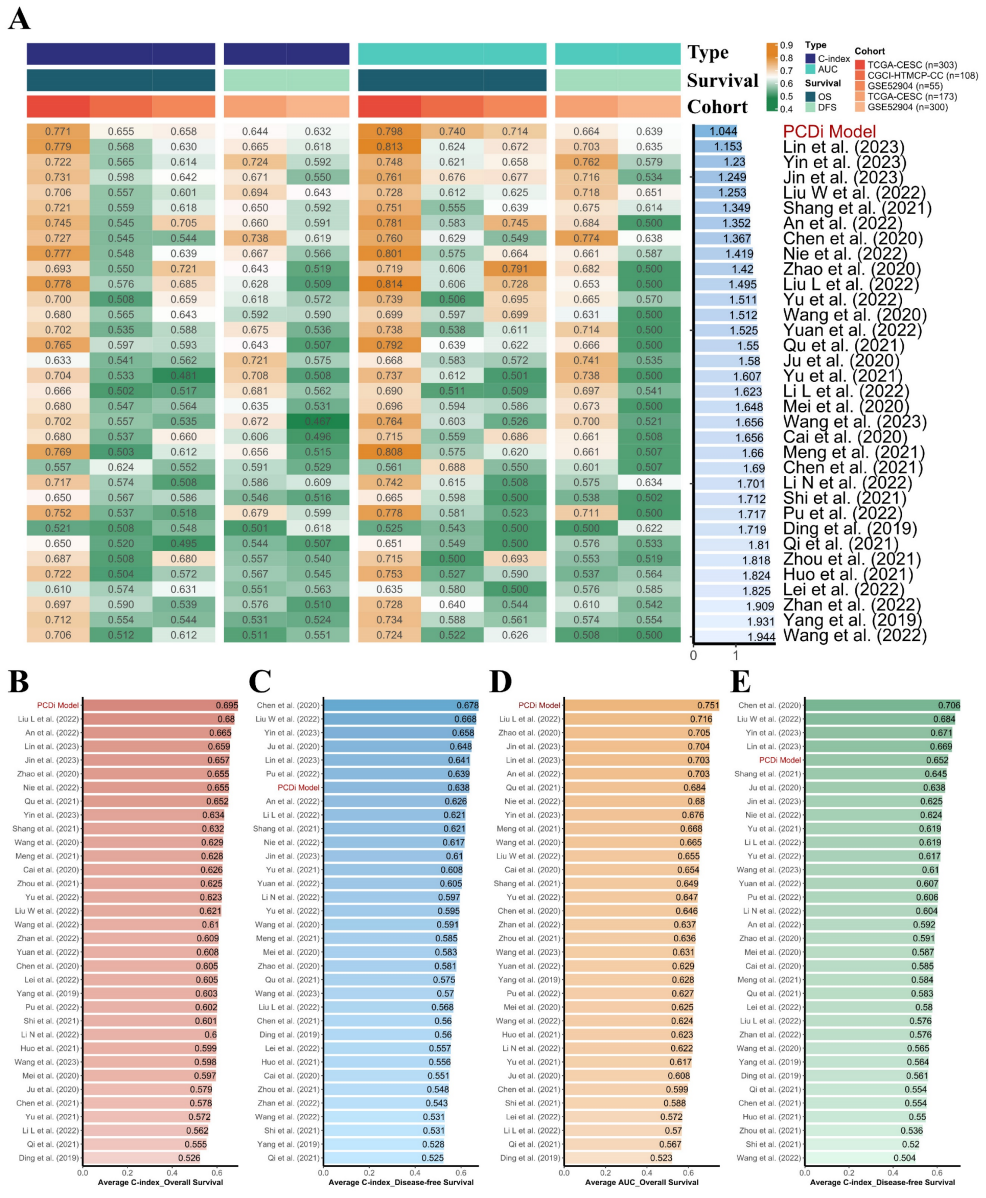
Comparison of the PCD-related prognostic signature and previously published signatures. (A) Comparison of the PCD-related prognostic signature and 33 published signatures for cervical cancer. The C-index and AUC values of each dataset were displayed, and the Rank Score was calculated for each signature based on the rank average and the number of genes in the model. (B) Average C-index of OS was calculated for each signature. (C) Average C-index of DFS was calculated for each signature. (D) Average AUC value of OS was calculated for each signature. (E) Average AUC value of DFS was calculated for each signature.

To evaluate the quality of the PCD-related prognostic signature, a total of 33 published mRNA-based prognostic signature for cervical cancer were retrieved through literature search for a comprehensive comparison (Figure 4, Supplementary Table S4). It is noteworthy that the PCD-related prognostic signature exhibited superior performance compared to other published signatures in the prognostic prediction for both OS and disease-free survival (DFS) in cervical cancer patients.

### Prognosis prediction by gene signature in training cohort and validation cohort

To validate our findings, we compared OS between cervical cancer patients with different PCDi scores in CGCI-HTMCP-CC and GSE52904 datasets. Cervical cancer patients with DFS data were also obtained from TCGA-CESC and GSE44001 datasets for validation.

**Figure 5:**
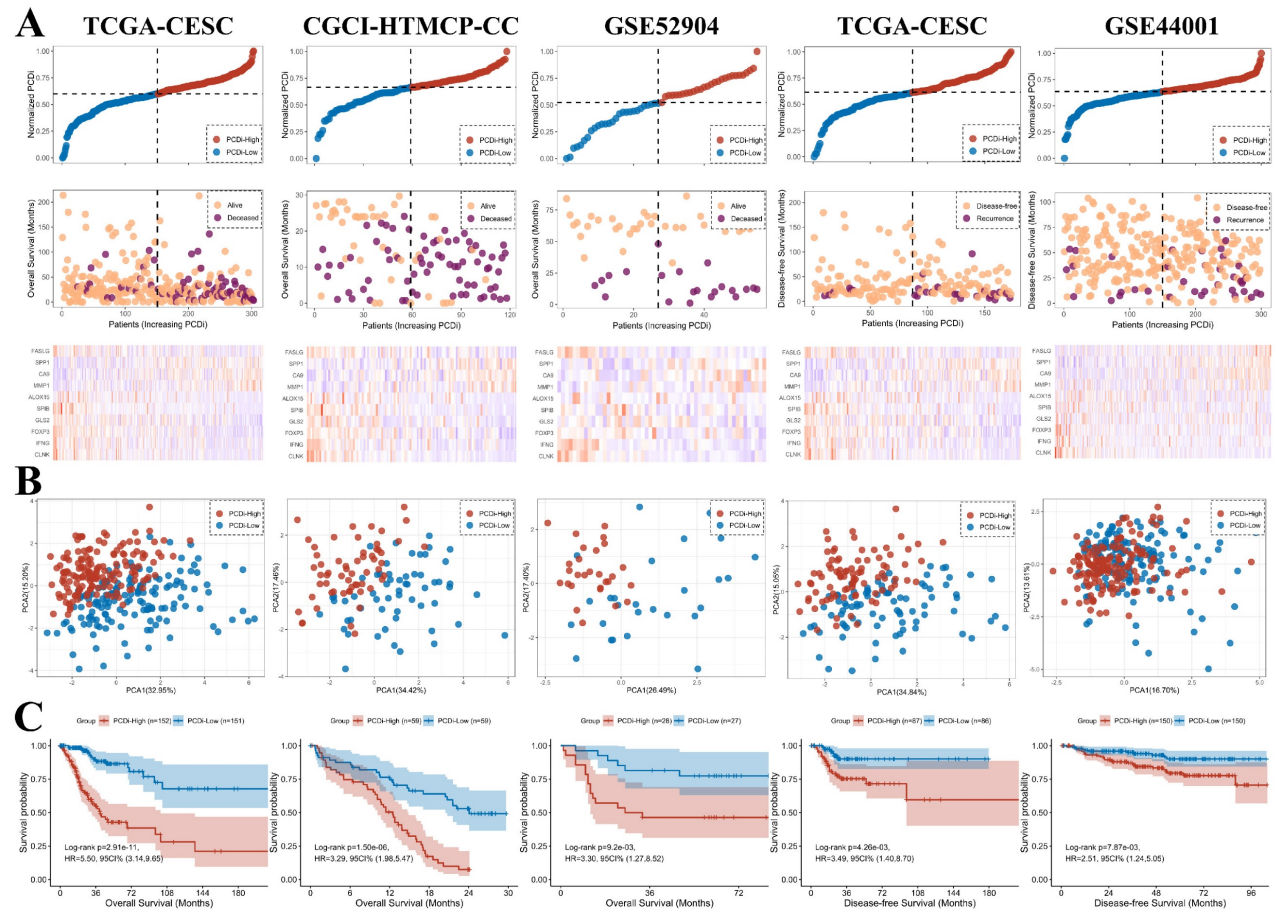
Validation of the PCD-related prognostic gene signature for cervical cancer. (A) Distribution of survival time and status of patients based on increasing normalized PCDi (from left to right) in TCGA-CESC, CGCI-HTMCP-CC, GSE52904, TCGA-CESC (DFS), and GSE44001 (DFS) datasets. (B) Expression levels of 10 PCD-related genes in patients with increasing normalized PCDi. (C) Principle component analysis (PCA) plot of patients based on the expression levels of 10 PCD-related genes. (D) KM estimates of OS in PCDi-High and PCDi-Low group patients.

The PCDi scores were normalized for better comparisons between datasets. Consistent with the results from TCGA-CESC dataset, higher death rate in cervical cancer patients with higher PCDi scores was observed in other datasets (Figure 5A). PCA plot revealed that the cervical cancer patients were well-separated based on PCDi (Figure 5B). The significant OS differences between PCDi-High and PCDi-Low subgroups were observed in both datasets from the training cohort. More importantly, this finding was further validated in the independent cervical cancer datasets, with significant survival differences in both OS and DFS (Figure 5C).

### PCDi-based nomogram survival model for cervical cancer

**Figure 6:**
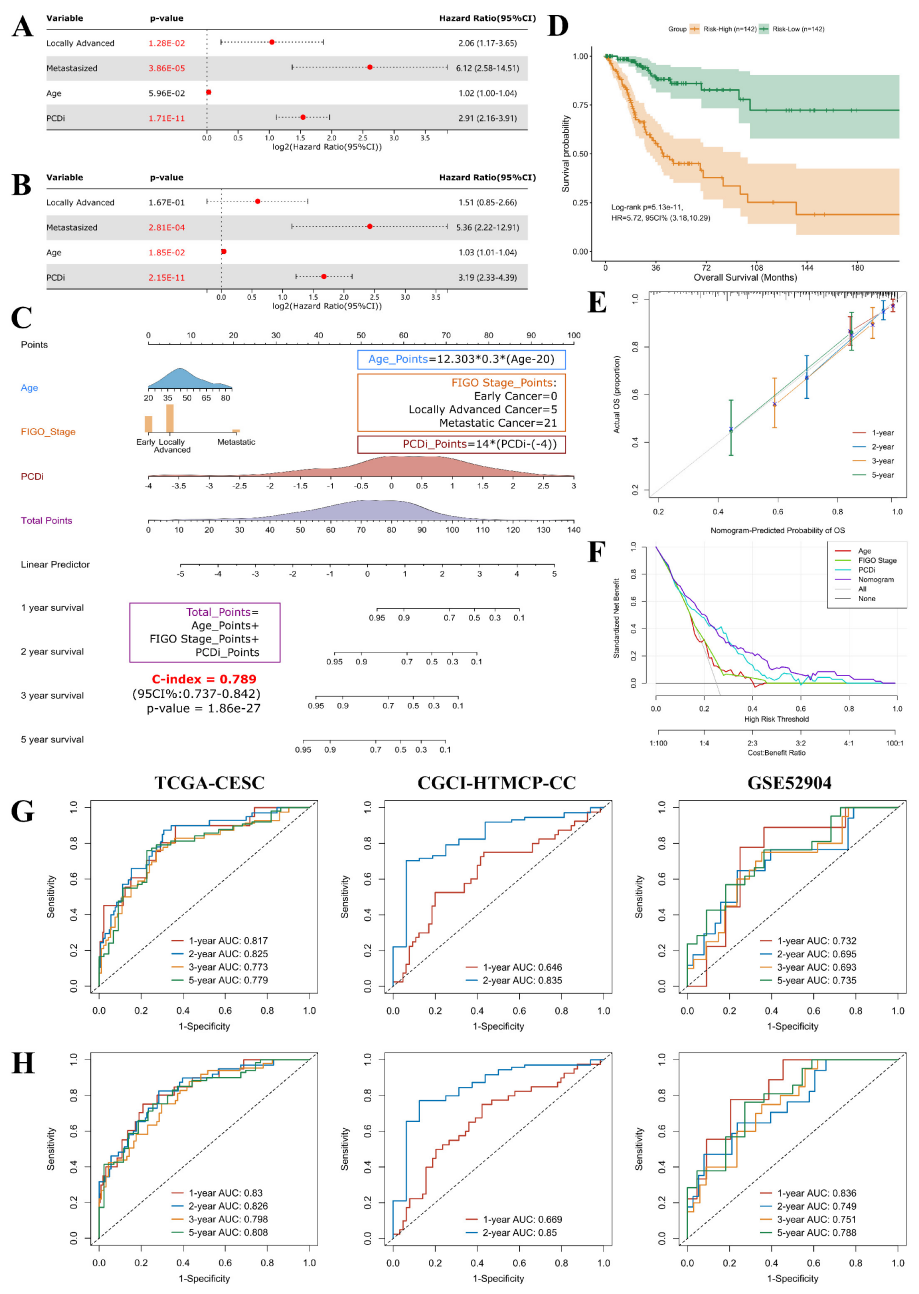
Development and assessment of the nomogram survival model for cervical cancer. (A) Univariate Cox regression analysis for PCDi and clinical data in TCGA-CESC dataset. The variable with a p-value <0.05 is highlighted in red. (B) Multivariate Cox regression analysis for PCDi and clinical data in TCGA-CESC dataset. The variable with a p-value <0.2 is kept in the regression model. The variable with a p-value <0.05 is highlighted in red. (C) Nomogram survival model developed by integrating PCDi and clinical characteristics. (D) KM estimate of OS in Risk-High and Risk-Low group patients classified by nomogram model. (E) Calibration plot of the nomogram model in predicting multi-year OS. The x-axis and y-axis indicate nomogram-predicted probability and actual probabil- ity of OS respectively. The 45-degree line represents ideal prediction, where the predicted probability matches the actual probability. The dots represent the prediction sets for different follow-up periods. The vertical line of each dot represents 95% CI of the actual probability. (F) DCA plot evaluating the performance of different predictors in predicting survival status. The x-axis and y-axis indicate the risk threshold and the standardized net benefit, respectively. The curves show the net benefit of using different predictors including Age (red), FIGO Stage (green), PCDi (cyan), and Nomogram (purple) across different threshold probabilities. A predictor with highest net benefit across a range of risk threshold is recommended. (G-H) Time-dependent ROC analysis evaluating the performances of PCD-related gene signature (G) and nomogram model (H) in predicting multi-year OS in TCGA- CESC, CGCI-HTMCP-CC, and GSE52904 datasets.

Prognostic factors for cervical cancer were identified using univariate Cox regression analysis. Our finding revealed that patients with high PCDi scores (Hazard Ratio = 2.91, 95% Confidence Interval: 2.16-3.91), as well as those with locally advanced stage (Stage IB2-IVA, HR = 2.06, 95% CI: 1.17-3.65) or metastasized stage (Stage IVB, HR = 6.12, 95% CI: 2.58-14.51) cervical cancer had higher risk of OS (Figure 6A).

To eliminate false discovery results contributed by the confounding factors, multivariate Cox analysis was performed, which confirmed that PCDi was an independent predictor of OS (HR = 3.19, 95% CI: 2.33-4.39, Figure 6B), highlighting its clinical significance in predicting the prognosis of cervical cancer patients (Supplementary Fig. S9). Based on multivariate Cox regression analysis, a nomogram survival model that integrated clinical features and PCDi was established to estimate 1-, 2-, 3-, and 5-year OS for cervical cancer patients (C-index = 0.789, 95% CI: 0.737-0.842, Figure 6C). High accuracy of the nomogram model in predicting the prognosis of cervical cancer patients was presented in Figure 6E. Additionally, DCA plot showed the nomogram survival model outperformed other predictors in the prediction of survival outcome (Figure 6F). Patients were categorized into Risk-High and Risk-Low subgroups based on the median score given by the nomogram survival model, revealing a significant survival difference (HR = 5.72, 95% CI: 3.18-10.29, Figure 6D). Furthermore, time-dependent ROC analysis demonstrated that the nomogram survival model had better performance than the PCD-related gene signature model in predicting multi-year OS of cervical cancer patients (Figure 6G and H). Our results highlight the clinical significance of PCDi as a prognostic factor and the utility of the nomogram survival model in the prediction of prognosis for cervical cancer.

### Immune features and drug sensitivities for cervical cancer

**Figure 7:**
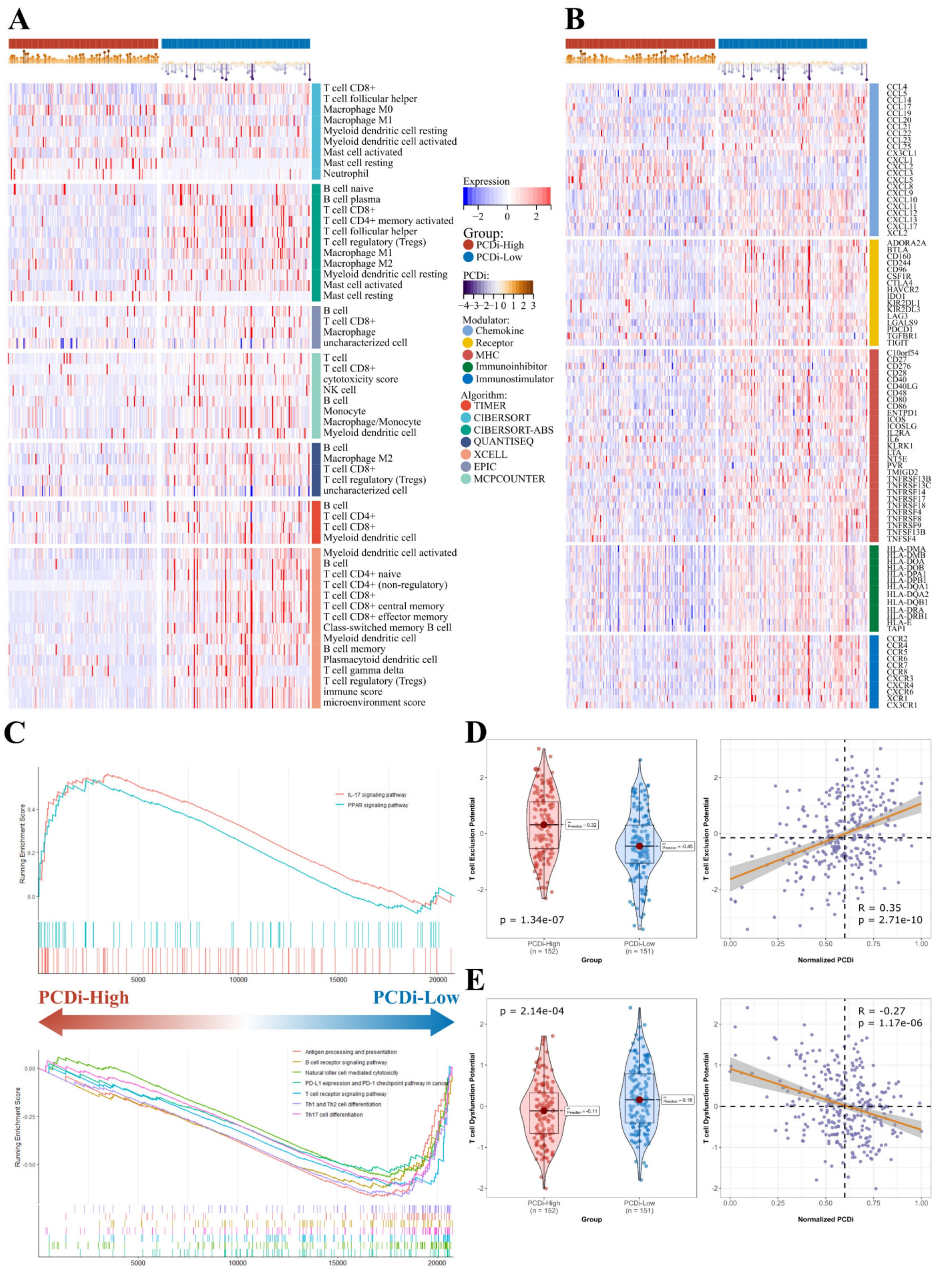
TME in cervical cancer patients with different PCDi scores. (A) Heatmap displaying the expression profiles of different tumor immune infiltrates between PCDi-High and PCDi-Low groups for cervical cancer. (B) Heatmap displaying the expression profiles of immune modulators between PCDi-High and PCDi-Low groups for cervical cancer. (C) Dysregulated signaling pathways identified in PCDi-High and PCDi-Low groups for cervical cancer. (D) Violin and scatter plots of the association between PCDi and T cell exclusion potential. (E) Violin and scatter plots of the association between PCDi and T cell dysfunction potential.

To investigate the differences in tumor immune features between two PCDi groups in cervical cancer, we estimated immune infiltrate abundance by calculating the enrichment scores of tumor-infiltrating immune cells using eight different algorithms (TIMER, CIBERSORT, CIBERSORT-ABS, EPIC, ESTIMATE, MCP-COUNTER, QUANTISEQ, and XELL). Additionally, the abundance of 28 tumor immune infiltrates was quantified using ssGSEA algorithm. Interestingly, our results revealed significant differences in tumor microenvironment (TME) of cervical cancer, with patients having higher PCDi scores exhibiting significantly lower tumor infiltration levels of immune cells (Figure 7A, Supplementary Fig. S10). Furthermore, most immune modulators were negatively correlated with PCDi, demonstrating a similar trend with TMB profiles (Figure 7B).

We utilized GSEA algorithm to further investigate the dysregulated signaling pathways between two PCDi groups. The results showed that immune-related signaling pathways were up-regulated in PCDi-Low group, such as antigen processing and presentation, B cell receptor signaling pathway, T cell receptor signaling pathway, and so on (Figure 7C). Additionally, we evaluated the potential for tumor immune escape and response using TIDE algorithm, which showed a higher potential of T cell dysfunction was observed in PCDi-Low group (Supplementary Fig. S11A and C), while the prediction scores of T cell exclusion were positively correlated with PCDi (Figure 7D and E, Supplementary Fig. S11B and D).

**Figure 8:**
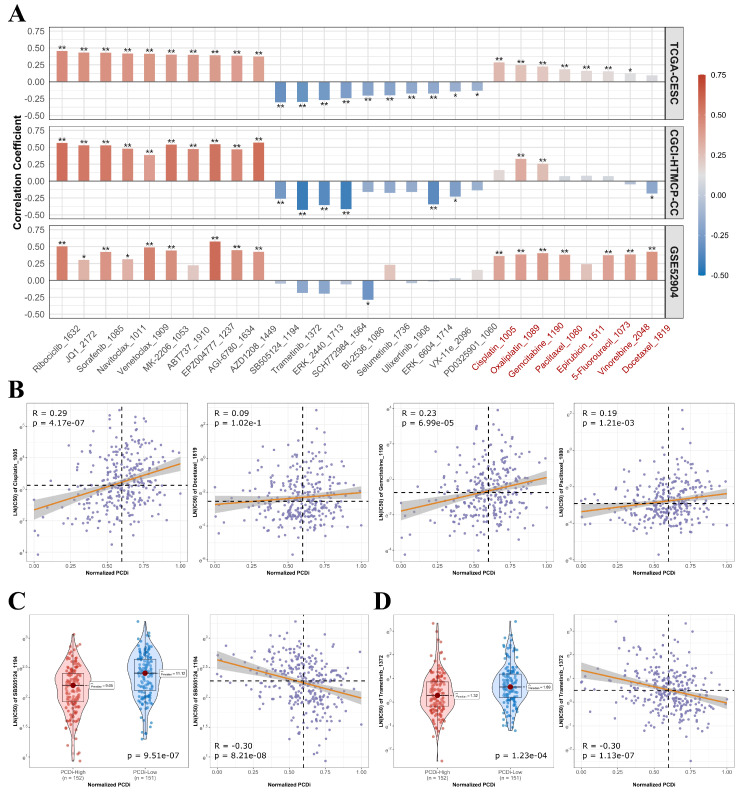
Drug sensitivities in cervical cancer patients with different PCDi scores. (A) Bar plot of the association between PCDi and IC50 value of different drugs. Commonly used chemotherapy regimens and platinum-based drugs are highlighted in red. Statistical significance is denoted by ** (p-value<0.01) and * (p-value <0.05). (B) Scatter plot of the association between PCDi and four commonly used chemotherapy regimens. (C-D) Violin and scatter plots of the association between PCDi and inhibitor, including SB505124 (C), and Trametinib (D).

Furthermore, we analyzed the association between drug's half maximum inhibitory concentration (IC50) and PCDi to predict the sensitivities of various drugs for cervical cancer (Figure 8A). We observed significant positive correlations between PCDi and IC50 values of commonly used chemotherapy regimens for cervical cancer such as cisplatin, docetraxel, paclitaxel, and gemcitabine (Figure 8B and C), indicating that patients with high PCDi scores were more likely resistant to chemotherapy. Fortunately, we also found that treatment options were available for PCDi-high group, such as SB505124 and Trametinib (Figure 8D and E), proving potential alternative therapeutic options for these patients.

### Experimental validation of MMP1

**Figure 9:**
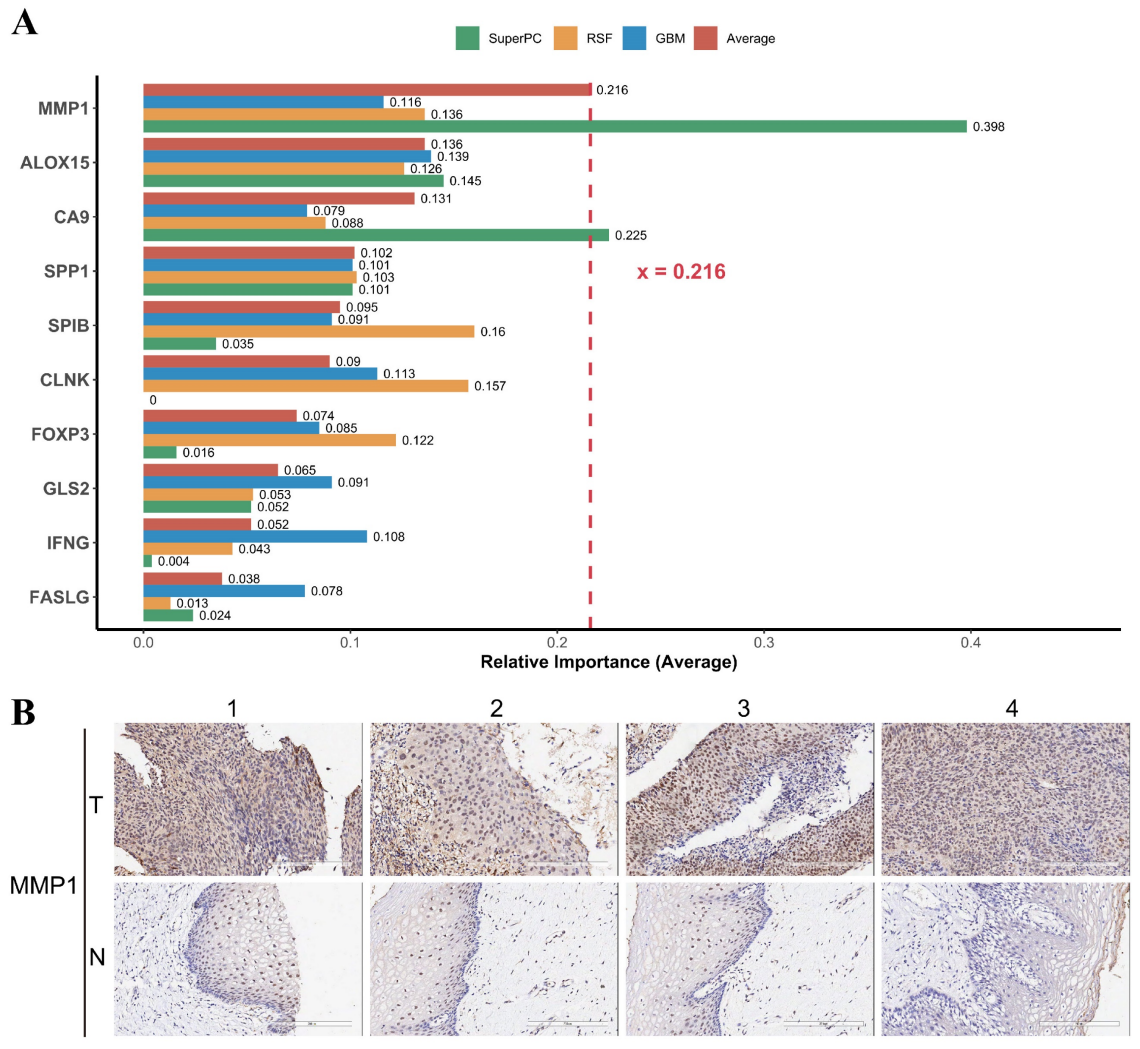
MMP1 is the most important gene in the PCD-related prognostic signature and is highly expressed in cervical cancer tissues. (A) Relative importance of individual genes to the PCD-related prognostic signature was calculated by three algorithms, including SuperPC (green), RSF (yellow), and GBM (blue). The red bar represents the average relative importance across the three algorithms. MMP1 had the highest average relative importance, indicating that it was the most influential gene in the prognostic signature. (B) Representative IHC staining images for MMP1 in cervical cancer tissues and normal tissues. The expression level of MMP1 was indicated by the intensity of the brown color. Cervical cancer tissues showed higher expression level of MMP1 than normal tissues, suggesting that MMP1 may play a role in the progression and invasion of cervical cancer.

For the developed PCD-related prognostic gene signature, we were interested in the contribution of individual genes to the signature. We calculated the relative importance of individual genes of our prognostic gene signature using three machine learning algorithm RSF, GBM, and SuperPC. We found that the relative importance of MMP1 gene to our prognostic gene signature was up 0.216 (Figure 9A). Matrix metalloproteinase-1 is encoded by gene MMP1, also known as interstitial collagenase, involves in the breakdown of interstitial collagens. It has been reported that the MMP1 was up-regulated and affecting lymph node metastasis of cervical cancer through PPAR signaling pathways *in vivo* 61.

According to GEPIA2 database (http://gepia2.cancer-pku.cn), the expression of MMP1 was significantly higher in the tumor tissue among different cancer types when comparing to the normal tissue (Supplementary Fig. 12). IHC staining images have shown that the overexpression of MMP1 in tumor tissue compared to normal tissue (Figure 9B). In short, the expression of MMP1 was elevated and may serve as a prognostic marker in cervical cancer.

## Discussion

To the best of our knowledge, this study represents the first comprehensive analysis of PCD patterns in TCGA-CESC dataset to develop a high-quality prognostic gene signature for cervical cancer. We introduced a novel concept named Rank Score to evaluate the performance of gene signature in prognostic prediction of cervical cancer. The calculation of a Rank Score of a gene signature not only integrated the prediction performance of OS and DFS, but also considered the number of genes included. The optimal signature model with very low Rank Score indicated its high C-index and AUC value in prognosis prediction and a reasonable number of genes. Our analysis resulted in a highly accurate OS prediction model that can aid in therapeutic decision-making, outperforming previously published prognostic signatures and validated by independent datasets. We further established a nomogram survival model that integrates PCDi and clinical features, demonstrating excellent performance in prognosis prediction. The FIGO staging system is one of the most important clinical indicators of the cancers originated from female reproductive system which provides accurate assessment of cancer development for appropriate disease management and prognostication in clinical settings. Due to the heterogeneity of cancer, the patients who have the same clinical stage could be further stratified into subgroups with two prognostic outcomes with the help of PCDi (Supplementary Fig. 7). Additionally, our findings highlight significant correlations between PCDi and tumor immune features and drug sensitivities, indicating the potential of PCDi to inform treatment decisions, and have significant implications in clinical practice.

Our PCD-related prognostic gene signature includes 10 genes (SPP1, SPIB, MMP1, ALOX15, GLS2, CA9, IFNG, FOXP3, FASLG, CLNK) that were found to be independent prognostic factors for the OS in cervical cancer. Higher expression of SPP1, MMP1, and CA9 was associated with a poorer prognosis. Secreted Phospho- protein 1 is encoded by gene SPP1, also known as Osteopontin (OPN), which has significant function roles in cancer development, such as cell proliferation and survival 62. OPN splice variant OPN-c support anchorage-independent growth by inducing the expression of oxidoreductases to avoid anoikis 63, 64. As a component of NETs, highly expressed MMP1 can promote tumor growth and metastasis in breast cancer cells 65, 66. CA9 is considered as an endogenous tumor hypoxia marker for cervical cancer, and its overexpression can promote the migration of tumor cells 67, 68. Lower expression of SPIB, ALOX15, GLS2, IFNG, FOXP3, FASLG, and CLNK was associated with a poorer prognosis. The activation of ETS transcription factor SPIB has been shown to increases anoikis resistance *in vitro* 69. In gastric cancer, cancer- associated fibroblasts secreted exo-miR-522 directly targets arachidonate lipoxygenase 15 (ALOX15) to suppress ferroptosis 70. In hepatocellular carcinoma, glutamine syn- thases 2 (GLS2) acts as a tumor suppressor by promoting ferroptosis through the production of α-ketoglutarate-dependent lipid ROS 71. Interferon gamma, encoded by gene IFNG, can induce caspase dependent cell apoptosis in pancreatic cancer cells through the upregulation of procaspase-1 and interferon regulatory factor 1 72. Fork- head box P3 (FOXP3), also known as Scurfin, plays a role in immune responses by regulating the development of regulatory T cells 73. As a member of the tumor necrosis factor family, FAS/FASLG signaling pathways is triggered by the binding of and FASLG (also known as FASL) to induce cell apoptosis. According to *in vitro* and *in vivo* evidence, FASLG targeted gene therapy could suppress the tumor growth in head and neck cancer 74. CLNK, also known as MIST, is an adaptor protein related to SLP76 protein family that regulates multiple immunoreceptor signaling pathways in a LAT-(linker for activation T cells) dependent manner 75. Among the 10 genes, we found the highest relative importance of MMP1 in the prognostic model. Our IHC results of cervical cancer patients further validated the overexpression of MMP1 in tumor tissue compared to normal tissue.

Tumor microenvironment is a complex interplay of signaling molecules, structural elements such as extracellular matrix, and various types of cells including stromal cells, immune cells, and tumor cells 76. The dynamic interactions between these components have profound impacts on cell survival, tumor growth, local invasion, and metastasis 77. Immune cells, as a critical component of TME, have dichotomous functions of either suppressing tumor formation or promoting tumorigenesis 78. Tumor cells are under surveillance by the immune system and can be attacked by various immune cells, such as cytotoxic T cells (CD8+), which play a crucial role in killing tumor cells by recognizing tumor antigens and subsequently suppressing tumor growth 77, 78. Infiltrating B cells, on the other hand, are involved in antigen production, antigen presentation, and secretion of cytokines instead of directly targeting tumor cells 77. In this study, we found that the enrichment of B cells and CD8+ T cells was negatively correlated with PCDi in cervical cancer patient, indicating lower infiltration of these immune cells in PCDi-High group. A review study has reported that higher infiltration of immune cells, including B cells and CD8+ T cells, is associated with better prognosis 79. Consistent with previous findings, a worse prognosis in cervical cancer patients with higher PCDi scores was observed in this study. Additionally, TIDE analysis revealed higher potentials of T cell dysfunction and T cell exclusion in PCDi-High group, supporting the significant difference in tumor immune features between two PCDi subgroups. Furthermore, we investigated the sensitivities of various drugs for cervical cancer by analyzing the association between PCDi and IC50. Higher IC50 value of commonly used chemotherapy regimens such as cisplatin, docetaxel, paclitaxel, and gemcitabine, were found in PCDi-High group, indicating high chemotherapy resistance in cervical cancer patients with high PCDi scores, which could potentially explain their poor prognosis. However, there are still treatment options available for patients in the PCDi-High group who have high sensitivity to these drugs, such as SB505124 and Trametinib. SB505124 is a selective inhibitor which targets transformation growth factor beta type I (TGF-beta) receptors ALK4, ALK5, and ALK7 and inhibits downstream Smad signaling 80. Previous studies have shown that SB505124 can restrain the migration and invasion of breast cancer cells 81. Trametinib, an FDA approved mitogen-activated protein kinase kinase (MEK) inhibitor, is used to treat melanoma patients with BRAF V600E. A phase II clinical trial is currently underway to evaluate the efficacy of combining Trametinib and AKT inhibitor GSK21411795 for the treatment of recurrent cervical cancer with PIK3CA and KRAS mutation 82.

In this study, we observed excellent performance of the PCD-related gene signature and the nomogram survival model in both training and validation cohorts. However, there are two limitations that should be acknowledged. Firstly, all tumor samples analyzed were retrospectively recruited, additional datasets with larger sample sizes, high quality, and longer follow-up periods will be required for further validation. Secondly, while our study highlights the importance of PCD-related genes as prognostic markers, there is still insufficient knowledge about some of these genes. Further research, particularly *in vivo* experiment, is warranted to better understand their roles in cervical cancer.

## Conclusions

In conclusion, we proposed a novel prognostic gene signature related to PCD for cervical cancer patients using a machine learning-based framework that can stratify them into two groups with significant differences in prognosis, tumor immune features, and drug sensitivity. Our signature model outperformed than previously published gene signatures in predicting patient's prognosis and demonstrated robustness and reproducibility in both training and validation cohorts. This signature model could serve as a valuable biomarker for identifying high-risk patients who may require more intensive treatment options or frequent disease surveillance. Moreover, we developed a nomogram survival model that integrates PCD-related gene signature and clinical characteristics to enhance the clinical utility of our signature model. Overall, our study highlights the potential usefulness of PCD-associated genes in predicting cervical cancer prognosis and emphasizes the importance of personalized treatment approaches tailored to individual patient characteristics. Our findings could have important implications in improving the management of cervical cancer patients.

## Supplementary Material

Supplementary methods, figures and table legends.

Supplementary tables.

## References

[1] World Health Organization. Fact Sheets of Cervical Cancer.

[2] Stelzle D, Tanaka LF, Lee KK (2021). Esti- mates of the Global Burden of Cervical Cancer Associated with HIV. The Lancet Global Health.

[3] Burk RD, Chen Z, Saller C (2017). Integrated Genomic and Molecular Characterization of Cervical Cancer. Nature.

[4] Yin M, Weng Y (2023). Eight Aging-Related Genes Prognostic Signature for Cervical Cancer. International Journal of Genomics.

[5] Liu W, Jiang Q, Sun C (2022). Developing a 5- Gene Prognostic Signature for Cervical Cancer by Integrating MRNA and Copy Number Variations. BMC Cancer.

[6] Lin Y, Zhang R, Pan H (2023). A Novel Immune-Related Signature to Predict Prognosis and Immune Infiltration of Cervical Cancer. Medical Science Monitor.

[7] Tang D, Kang R, Berghe TV (2019). The Molecular Machinery of Regulated Cell Death. Cell Research.

[8] Ameisen JC (2002). On the Origin, Evolution, and Nature of Programmed Cell Death: A Timeline of Four Billion Years. Cell death and differentiation.

[9] Wong RSY (2011). Apoptosis in Cancer: From Pathogenesis to Treatment. Journal of experimental and clinical cancer research.

[10] Pfeffer CM, Singh ATK (2018). Apoptosis: A Target for Anticancer Therapy. International Journal of Molecular Sciences.

[11] Carneiro BA, El-Deiry WS (2020). Targeting Apoptosis in Cancer Therapy. Nature Reviews Clinical Oncology.

[12] Sperandio S, Poksay K, de Belle I (2004). Paraptosis: Mediation by MAP Kinases and Inhibition by AIP-1/Alix. Cell Death and Differentiation.

[13] Fontana F, Raimondi M, Marzagalli M (2020). The Emerging Role of Paraptosis in Tumor Cell Biology: Perspectives for Cancer Prevention and Therapy with Natural Compounds. Biochimica et Biophysica Acta - Reviews on Cancer.

[14] Wang F, Gómez-Sintes R, Boya P (2018). Lysosomal Membrane Permeabilization and Cell Death. Traffic.

[15] Aits S, Jäättelä M (2013). Lysosomal Cell Death at a Glance. Journal of Cell Science.

[16] Repnik U, Česen MH, Turk, B (2014). Lysosomal Membrane Permeabilization in Cell Death: Concepts and Challenges. Mitochondrion.

[17] Yu P, Zhang X, Liu N (2021). Pyroptosis: Mechanisms and Diseases. Signal Transduction and Targeted Therapy.

[18] Tan Y, Chen Q, Li X (2021). Pyroptosis: A New Paradigm of Cell Death for Fighting against Cancer. Journal of Experimental and Clinical Cancer Research.

[19] Vorobjeva NV, Chernyak BV (2020). NETosis: Molecular Mechanisms, Role in Physiology and Pathology. Biochemistry (Moscow).

[20] Kroemer G, Galassi C, Zitvogel L (2022). Immunogenic Cell Stress and Death. Nature Immunology.

[21] Krysko DV, Garg AD, Kaczmarek A (2012). Immunogenic Cell Death and DAMPs in Cancer Therapy. Nature Reviews Cancer.

[22] Dhuriya YK, Sharma D (2018). Necroptosis: A Regulated Inflammatory Mode of Cell Death. Journal of Neuroinflammation.

[23] Galluzzi L, Kepp O, Chan FKM (2017). Necroptosis: Mechanisms and Relevance to Disease. Annual Review of Pathology: Mechanisms of Disease.

[24] Kianfar M, Balcerak A, Chmielarczyk M (2022). Cell Death by Entosis: Triggers, Molecular Mechanisms and Clinical Significance. International Journal of Molecular Sciences.

[25] Fatokun AA, Dawson VL, Dawson TM (2013). Parthanatos: Mitochondrial- Linked Mechanisms and Therapeutic Opportunities. Br J Pharmacol.

[26] Taddei ML, Giannoni E, Fiaschi T (2012). Anoikis: An Emerging Hallmark in Health and Diseases. Journal of Pathology.

[27] Zhang C, Liu X, Jin S (2022). Ferroptosis in Cancer Therapy: A Novel Approach to Reversing Drug Resistance. Molecular Cancer.

[28] Li J, Cao F, Yin H (2020). Ferroptosis: Past, Present and Future. Cell Death and Disease.

[29] Jiang X, Stockwell BR, Conrad M (2021). Ferroptosis: Mechanisms, Biology and Role in Disease. Nature Reviews Molecular Cell Biology.

[30] Denton D, Kumar S (2019). Autophagy-Dependent Cell Death. Cell Death and Differentiation.

[31] Liu J, Kuang F, Kang R (2020). Alkaliptosis: A New Weapon for Cancer Therapy. Cancer Gene Therapy.

[32] Scaturro P, Pichlmair A (2019). Oxeiptosis: A Discreet Way to Respond to Radicals. Current Opinion in Immunology.

[33] Tang D, Chen X, Kroemer G (2022). Cuproptosis: A Copper-Triggered Modality of Mitochondrial Cell Death. Cell Research.

[34] Wang Y, Zhang L, Zhou F (2022). Cuproptosis: A New Form of Pro- grammed Cell Death. Cellular and molecular immunology.

[35] Wang L, Qin X, Liang J (2021). Induction of Pyroptosis: A Promising Strategy for Cancer Treatment. Frontiers in Oncology.

[36] Adeshakin FO, Adeshakin AO, Afolabi LO (2021). Mechanisms for Modulating Anoikis Resistance in Cancer and the Relevance of Metabolic Reprogramming. Frontiers in Oncology.

[37] Prateep A, Sumkhemthong S, Karnsomwan W (2018). Avicequinone B Sensitizes Anoikis in Human Lung Cancer Cells. Journal of Biomedical Science.

[38] Zhang M, Hong X, Ma N (2023). The Promoting Effect and Mechanism of Nrf2 on Cell Metastasis in Cervical Cancer. Journal of Translational Medicine.

[39] Mohanty S, Yadav P, Lakshminarayanan H (2022). RETRA Induces Necroptosis in Cervical Cancer Cells through RIPK1, RIPK3, MLKL and Increased ROS Production. European Journal of Pharmacology.

[40] Jaudan A, Sharma S, Malek SNA (2018). Induction of Apoptosis by Pinostrobin in Human Cervical Cancer Cells: Possible Mechanism of Action. PLoS One.

[41] Su Z, Yang Z, Xu Y (2015). Apoptosis, Autophagy, Necroptosis, and Cancer Metastasis. Molecular Cancer.

[42] Zou Y, Xie J, Zheng S (2022). Leveraging Diverse Cell-Death Patterns to Predict the Prognosis and Drug Sensitivity of Triple-Negative Breast Cancer Patients after Surgery. International Journal of Surgery.

[43] Ye Y, Dai Q, Qi H (2021). A Novel Defined Pyroptosis-Related Gene Signature for Predicting the Prognosis of Ovarian Cancer. Cell Death Discovery.

[44] Garg AD, Ruysscher DD, Agostinis P (2016). Immunological Metagene Signatures Derived from Immunogenic Cancer Cell Death Associate with Improved Survival of Patients with Lung, Breast or Ovarian Malignancies: A Large-Scale Meta-Analysis. OncoImmunology.

[45] Stelzer G, Rosen N, Plaschkes I (2016). The GeneCards Suite: From Gene Data Mining to Disease Genome Sequence Analyses. Current protocols in bioinformatics.

[46] Zhou N, Yuan X, Du Q (2023). FerrDb V2: Update of the Manually Curated Database of Ferroptosis Regulators and Ferroptosis-Disease Associations. Nucleic acids research.

[47] Goldman MJ, Craft B, Hastie M Visualizing and Interpreting Cancer Genomics Data via the Xena Platform. Nature Biotechnology. 2020: 38: 675-678.

[48] Gagliardi A, Porter VL, Zong Z (2020). Analysis of Ugandan Cervical Carcinomas Identifies Human Papillomavirus Clade-Specific Epigenome and Transcriptome Landscapes. Nature Genetics.

[49] Medina-Martinez I, Barrón V, Roman-Bassaure E (2014). Impact of Gene Dosage on Gene Expression, Biological Processes and Survival in Cervical Cancer: A Genome-Wide Follow-up Study. PLoS ONE.

[50] Lee YY, Kim TJ, Kim JY (2013). Genetic Profiling to Predict Recurrence of Early Cervical Cancer. Gynecologic oncology.

[51] Love MI, Huber W, Anders S (2014). Moderated Estimation of Fold Change and Dispersion for RNA-Seq Data with DESeq2. Genome Biology.

[52] Ritchie ME, Phipson B, Wu D (2015). Limma Powers Differential Expression Analyses for RNA- Sequencing and Microarray Studies. Nucleic Acids Research.

[53] Robinson MD, McCarthy DJ, Smyth GK (2009). EdgeR: A Bioconductor Pack- age for Differential Expression Analysis of Digital Gene Expression Data. Bioinformatics.

[54] Yu G, Wang LG, Han Y (2012). ClusterProfiler: An R Package for Com- paring Biological Themes among Gene Clusters. OMICS A Journal of Integrative Biology.

[55] Supek F, Bošnjak M, Škunca N (2011). Revigo Summarizes and Visualizes Long Lists of Gene Ontology Terms. PLoS ONE.

[56] Li T, Fu J, Zeng Z (2020). TIMER2.0 for Analysis of Tumor-Infiltrating Immune Cells. Nucleic Acids Research.

[57] Jiang P, Gu S, Pan D (2018). Signatures of T Cell Dysfunction and Exclusion Predict Cancer Immunotherapy Response. Nature Medicine.

[58] Ru B, Wong CN, Tong Y (2019). TISIDB: An Integrated Repository Portal for Tumor-Immune System Interactions. Bioinformatics.

[59] Maeser D, Gruener RF, Huang RS (2021). OncoPredict: An R Package for Predicting *in vivo* or Cancer Patient Drug Response and Biomarkers from Cell Line Screening Data. Briefings in Bioinformatics.

[60] Chen P, Wang W, Wong S (2022). RUNDC3A Regulates SNAP25-Mediated Chemotherapy Resistance by Binding AKT in Gastric Neuroendocrine Carcinoma (GNEC). Cell Death Discov.

[61] Tian R, Li X, Gao Y (2018). Identification and Validation of the Role of Matrix Metalloproteinase-1 in Cervical Cancer. International Journal of Oncology.

[62] Shevde LA, Samant RS (2014). Role of Osteopontin in the Pathophysiology of Cancer. Matrix Biology.

[63] Shen H, Weber GF (2014). The Osteopontin-c Splice Junction Is Important for Anchorage-Independent Growth. Molecular Carcinogenesis.

[64] He B, Mirza M, Weber GF (2006). An Osteopontin Splice Variant Induces Anchorage Independence in Human Breast Cancer Cells. Oncogene.

[65] Liu H, Kato Y, Erzinger SA (2012). The Role of MMP-1 in Breast Cancer Growth and Metastasis to the Brain in a Xenograft Model. BMC Cancer.

[66] Shao BZ, Yao Y, Li JP (2021). The Role of Neutrophil Extracellular Traps in Cancer. Frontiers in Oncology.

[67] Hsin MC, Hsieh YH, Hsiao YH (2021). Carbonic Anhydrase Ix Promotes Human Cervical Cancer Cell Motility by Regulating Pfkfb4 Expression. Cancers.

[68] Olive PL, Aquino-Parsons C, MacPhail SH (2001). Carbonic Anhydrase 9 as an Endogenous Marker for Hypoxic Cells in Cervical Cancer. Cancer research.

[69] Zhang H, Wang G, Zhou R (2020). SPIB Promotes Anoikis Resistance via Elevated Autolysosomal Process in Lung Cancer Cells. FEBS Journal.

[70] Zhang H, Deng T, Liu R (2020). CAF Secreted MiR-522 Suppresses Ferroptosis and Promotes Acquired Chemo-Resistance in Gastric Cancer. Molecular Cancer.

[71] Suzuki S, Venkatesh D, Kanda H (2022). GLS2 Is a Tumor Suppressor and a Regulator of Ferroptosis in Hepatocellular Carcinoma. Cancer research.

[72] Detjen KM, Farwig K, Welzel M (2001). Interferon γ Inhibits Growth of Human Pancreatic Carcinoma Cells via Caspase-1 Dependent Induction of Apoptosis. Gut.

[73] Hori S, Nomura T, Sakaguchi S (2003). Control of Regulatory T Cell Development by the Transcription Factor Foxp3. Science.

[74] ElOjeimy S, McKillop JC, El-Zawahry AM (2006). FasL Gene Therapy: A New Therapeutic Modality for Head and Neck Cancer. Cancer Gene Therapy.

[75] Goitsuka R, Tatsuno A, Ishiai M (2001). MIST Functions through Distinct Domains in Immunoreceptor Signaling in the Presence and Absence of LAT. The Journal of biological chemistry.

[76] Baghban R, Roshangar L, Jahanban-Esfahlan R (2020). Tumor Microenvironment Complexity and Therapeutic Implications at a Glance. Cell Communication and Signaling.

[77] Neophytou CM, Panagi M, Stylianopoulos T (2021). The Role of Tumor Microenvironment in Cancer Metastasis: Molecular Mechanisms and Therapeutic Opportunities. Cancers.

[78] Anderson NM, Simon MC (2020). The Tumor Microenvironment. Current Biology.

[79] Barnes TA, Amir E (2017). HYPE or HOPE: The Prognostic Value of Infiltrating Immune Cells in Cancer. British Journal of Cancer.

[80] Byfield SD, Major C, Laping NJ (2004). SB-505124 Is a Selective Inhibitor of Transforming Growth Factor-Beta Type I Recep- tors ALK4, ALK5, and ALK7. Molecular pharmacology.

[81] Goto N, Hiyoshi H, Ito I (2011). Estrogen and Antiestrogens Alter Breast Cancer Invasiveness by Modulating the Transforming Growth Factor-β Signaling Pathway. Cancer Science.

[82] Liu JF, Gray KP, Wright AA (2019). Results from a Single Arm, Single Stage Phase II Trial of Trametinib and GSK2141795 in Persistent or Recurrent Cervical Cancer. Gynecologic oncology.

