# Supplementary material for: Programmed cell death-index (PCDi) as a prognostic biomarker and predictor of drug sensitivity in cervical cancer: a machine learning-based analysis of mRNA signatures
Source: J Cancer. 2024 Jan 20;15(5):1378–96. doi: 10.7150/jca.91798 (PMC10861809; doi:10.7150/jca.91798)
Supplement: Supplementary file 1 — Supplementary methods, figures and table legends. [file jcav15p1378s1.pdf]

# Supplementary Materials

Wei Wang

December 4, 2023

## 1 Supplementary Method

To develop the optimal gene signature, ten machine learning algorithms were employed in a 10-fold cross-validation framework to identify the optimal gene signature for cervical cancer, including survival support vector machine (survivalSVM), elastic network (Enet), least absolute shrinkage and selection operator (LASSO), ridge regression (Ridge), random survival forest (RSF), stepwise Cox regression analysis (stepwiseCox), fitting Cox models by likelihood based boosting (CoxBoost), generalized boosted regression modeling (GBM), partial least squares regression for Cox (plsRcox), and supervised principal components (SuperPC). Implementation of all machine learning algorithms was performed via R software (http://www.R-project.org). The R package "*randomForestSRC*" was utilized to perform RSF modeling with the parameter *ntree* and *nsplit* set to 1000 and 10, respectively. The variables with relative importance no less than 0.1 were kept in the model. The R package "*glmnet*" was used to perform Enet, LASSO, and Ridge regression models, with *lambda.min* value used in regression analysis. The parameter *alpha* was set within a range of 0.1 to 0.9 (interval=0.1) to identify the best model in Enet regression. The R package "*survival*" was used to perform stepwiseCox regression to yield the optimal model with the minimum AIC value. CoxBoost modeling was performed using the R package "*CoxBoost*" by likelihood based boosting with the parameter *start.penalty* set to 500. Least squares regression for Cox survival models was performed using the R package "*plsRcox*" with the parameter *nt* set to 20. The R package "*superpc*" was used to perform prognosis prediction using supervised principal component method. Generalized boosted regression was performed using the R package "*gbm*" with the parameter *n.tree* and *shrinkage* set to 1000 and 0.01, respectively. The variables with relative influence no less than 10 were kept in the regression model. Support vector analysis for data with survival outcomes was performed using the R package "*survivalsvm*" with *Vanbelle1* type of support vector model implemented and the parameter *gamma.mu* set to 1.

The R packages of machine learning algorithm:
https://cran.r-project.org/web/packages/randomForestSRC/index.html
https://cran.r-project.org/web/packages/glmnet/index.html
https://cran.r-project.org/web/packages/survival/index.html
https://github.com/binderh/CoxBoost
https://cran.r-project.org/web/packages/plsRcox/index.html
https://cran.r-project.org/web/packages/superpc/index.html
https://cran.r-project.org/web/packages/gbm/index.html
https://cran.r-project.org/web/packages/survivalsvm/index.html

# 2 Supplementary Tables and Figures

## 2.1 Supplementary Tables

Table S1. Genes associated to fifteen types of programmed cell death (PCD) patterns
Table S2. Identification of differentially expressed genes (DEGs) by three different algorithms
Table S3. Development of the PCD-related prognostic signature by machine learning algorithms
Table S4. Comparison of the PCD-related prognostic signature and previously published signatures

## 2.2 Supplementary Figures

Figure S1. Kaplan-Meier (KM) estimates of overall survival (OS) in cervical cancer patients with different FIGO cancer stages.
Figure S2. Sample inclusion and exclusion criteria of this study.
Figure S3. Univariate Cox regression analysis identified 27 PCD-related candidate genes.
Figure S4. Comparison of expression levels of 12 PCD-related genes in tumor and normal tissues.
Figure S5. KM estimates of OS in cervical cancer patients with different expression levels of 10 PCD-related genes.
Figure S6. Association between programmed cell death-index (PCDi) score and clinical features in cervical cancer patients.
Figure S7. KM estimates of OS in patients with different PCDi scores for a particular FIGO cancer stage.
Figure S8. KM estimates of OS in patients with different PCDi scores and FIGO cancer stage.
Figure S9. Multivariate Cox regression analysis in different study cohorts.
Figure S10. The expression profiles of different tumor immune infiltrates between PCDi-High and PCDi-Low groups through ssGSEA algorithm.
Figure S11. Violin and scatter plots displaying the association between PCDi and T cell dysfunction potential and exclusion potential.
Figure S12. MMP1 expression levels in different cancer types.

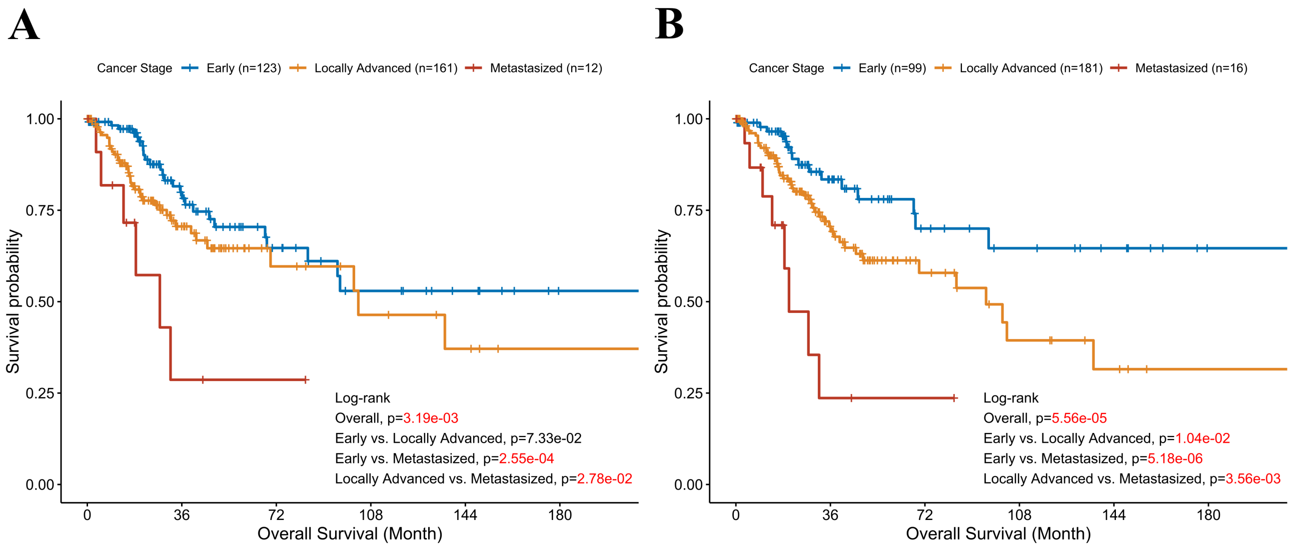


Figure S1. Kaplan-Meier (KM) estimates of overall survival (OS) in cervical cancer patients with different FIGO cancer stages. (A). Before conversion. (B). After conversion.

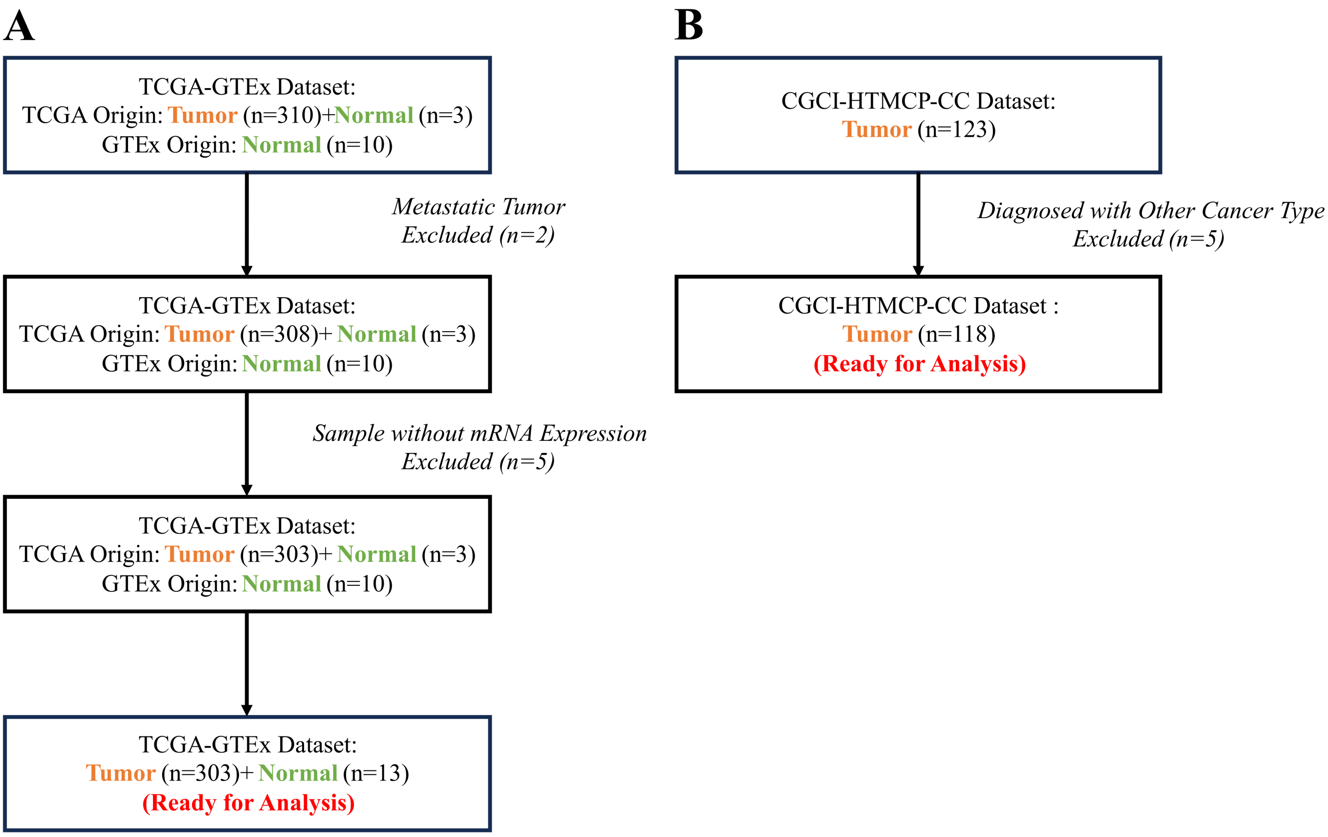


Figure S2. Sample inclusion and exclusion criteria of this study. (A). TCGA-GTEx dataset. (B). CGCI-HTMCP-CC dataset.

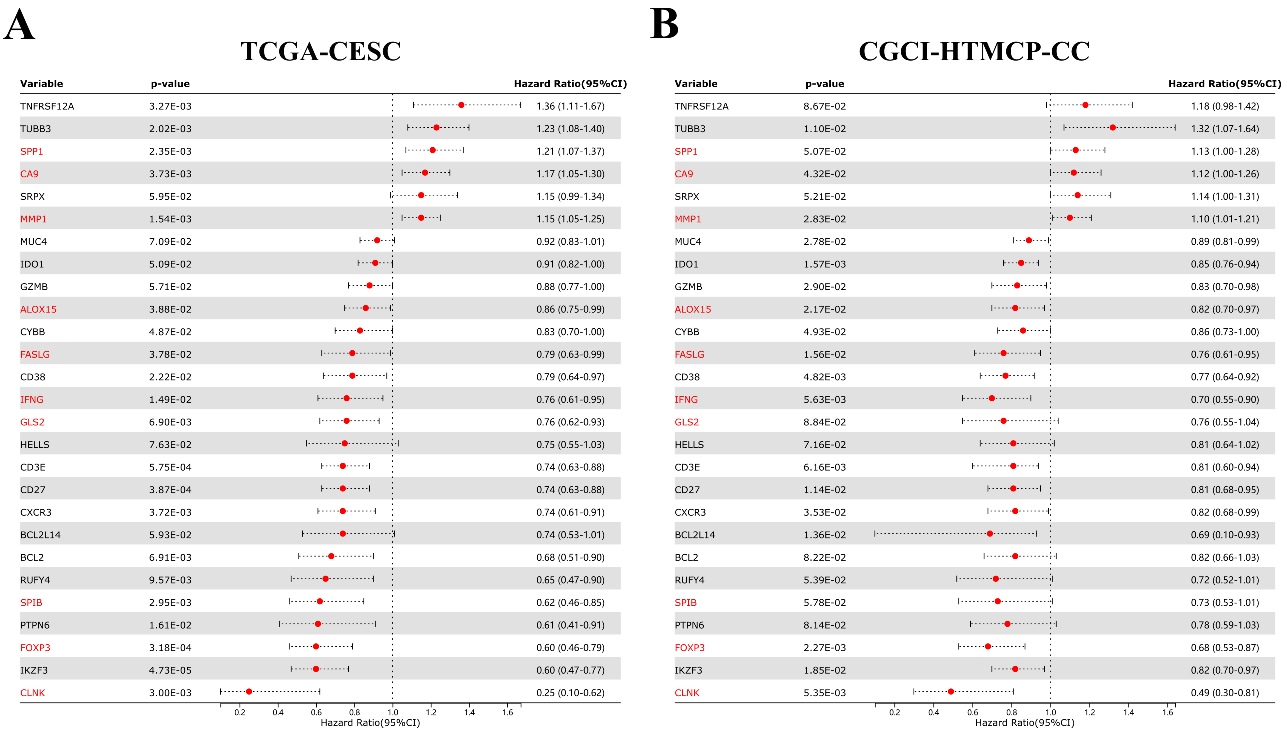


Figure S3. Univariate Cox regression analysis identified 27 PCD-related candidate genes. (A). TCGA-CESC dataset. (B). CGCI-HTMCP-CC dataset.

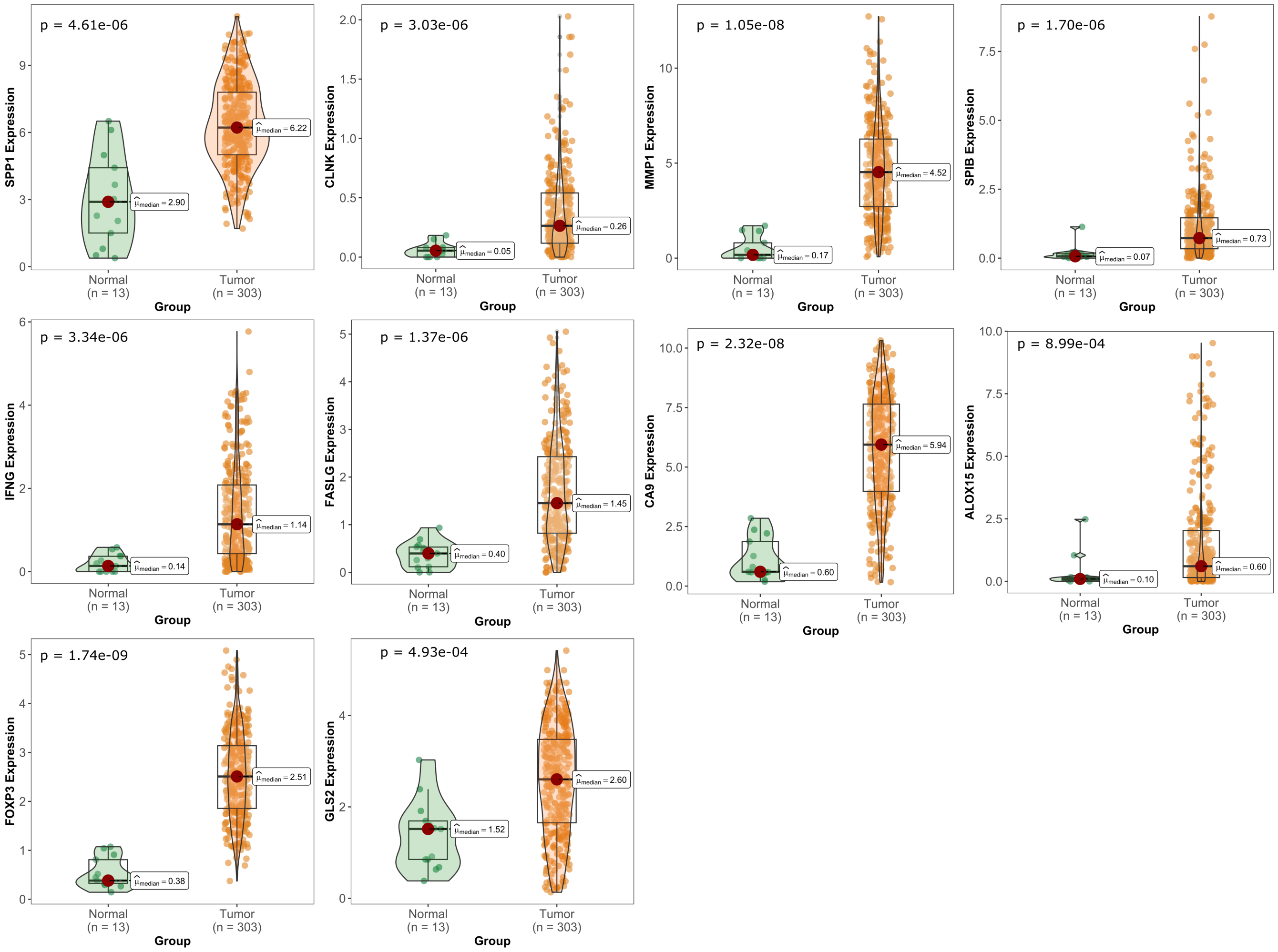


Figure S4. Comparison of expression levels of 12 PCD-related genes in tumor and normal tissues.

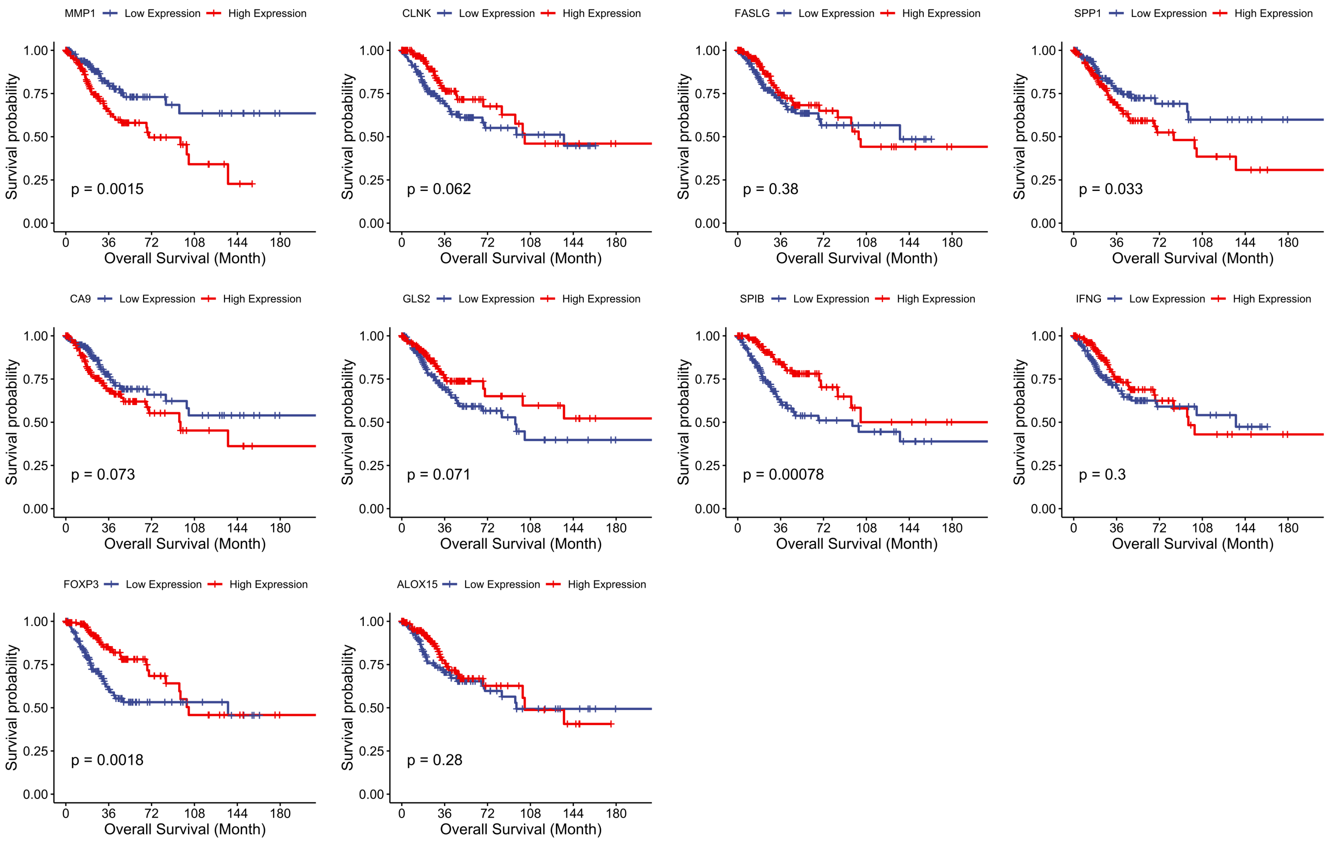


Figure S5. KM estimates of OS in cervical cancer patients with different expression levels of 10 PCD-related genes.

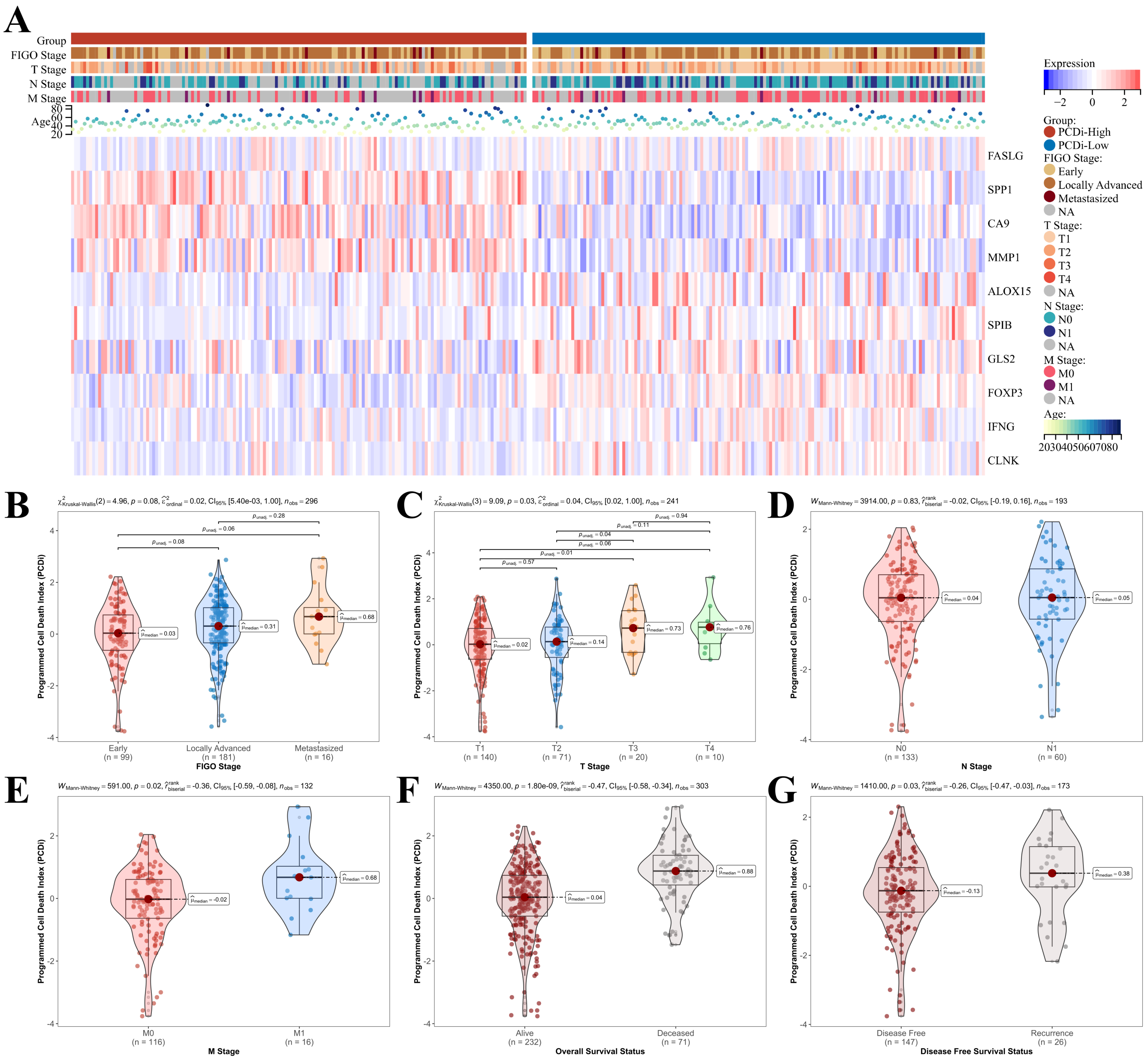


Figure S6. Association between programmed cell death-index (PCDi) score and clinical features in cervical cancer patients. (A). Heatmap displaying the expression levels of 10 PCD-related genes in relation to clinical characteristics. (B-G). Violin plots depicting the association between normalized PCDi and clinical characteristics, including FIGO cancer stage (B), T stage (C), N stage (D), M stage (E), overall survival status (F), and disease-free survival status (G).

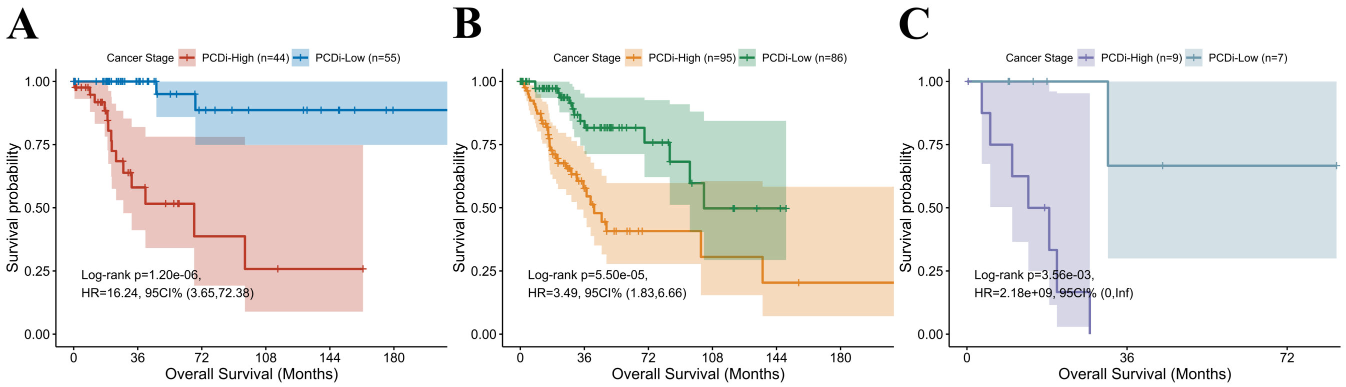


Figure S7. KM estimates of OS in patients with different PCDi scores for a particular FIGO cancer stage. (A). Early stage (<IB2). (B). Locally advanced stage (IB2-IVA). (C). Metastasized stage (IVB).

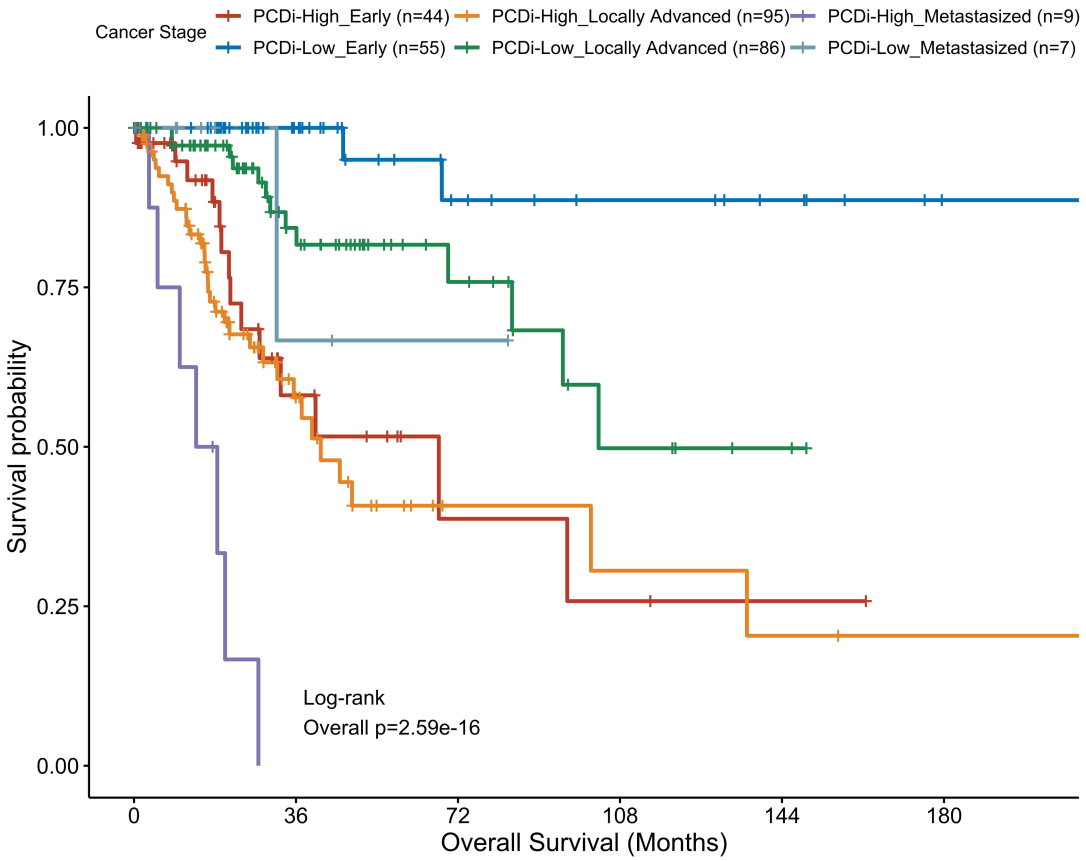


Figure S8. KM estimates of OS in patients with different PCDi scores and FIGO cancer stage.

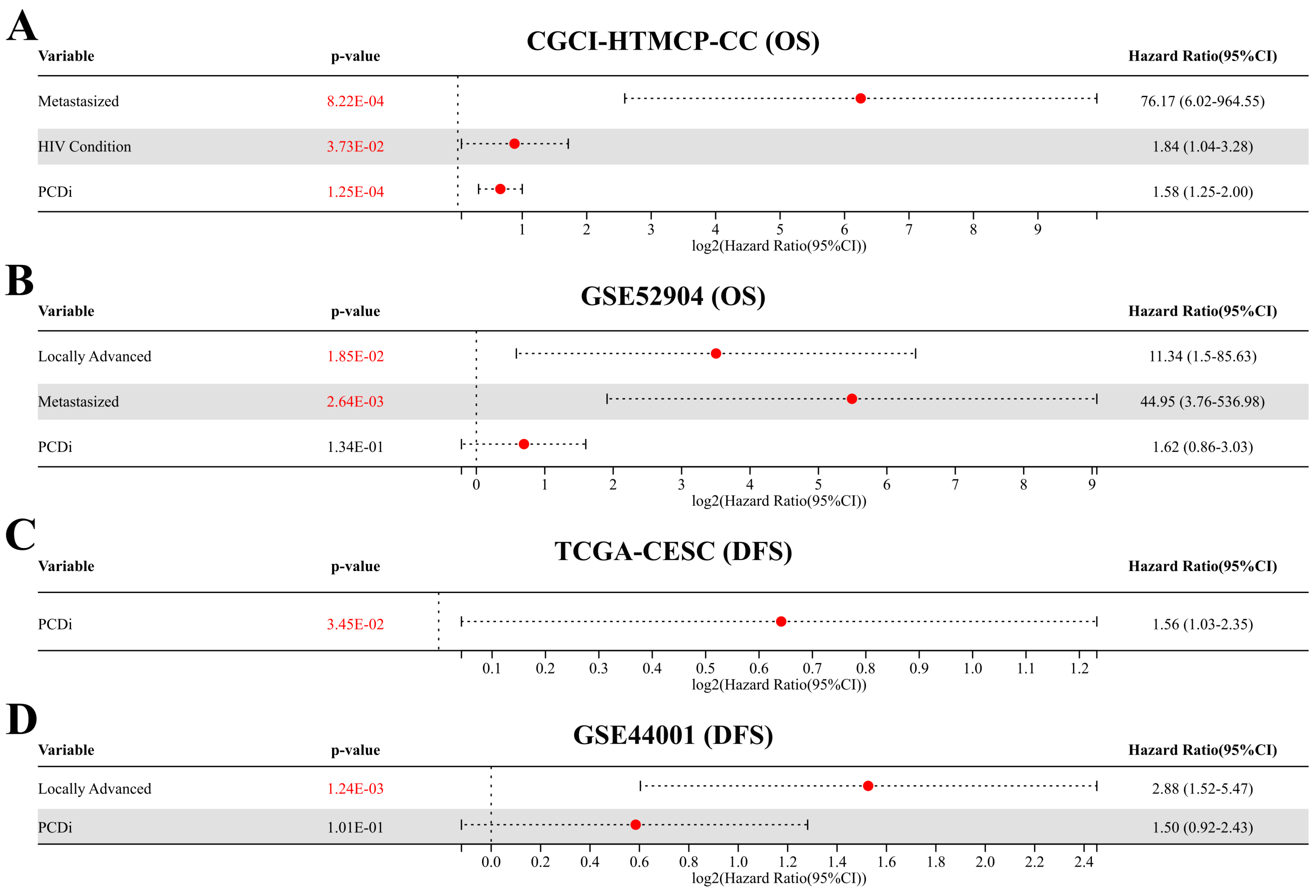


Figure S9. Multivariate Cox regression analysis in different study cohorts. The variable with a p-value ¡0.2 is kept in the regression model. The variable with a p-value ¡0.05 is highlighted in red. (A). CGCI-HTMCP-CC dataset (OS). (B). GSE52904 dataset (OS). (C). TCGA-CESC dataset (DFS). (D). GSE44001 dataset (DFS).

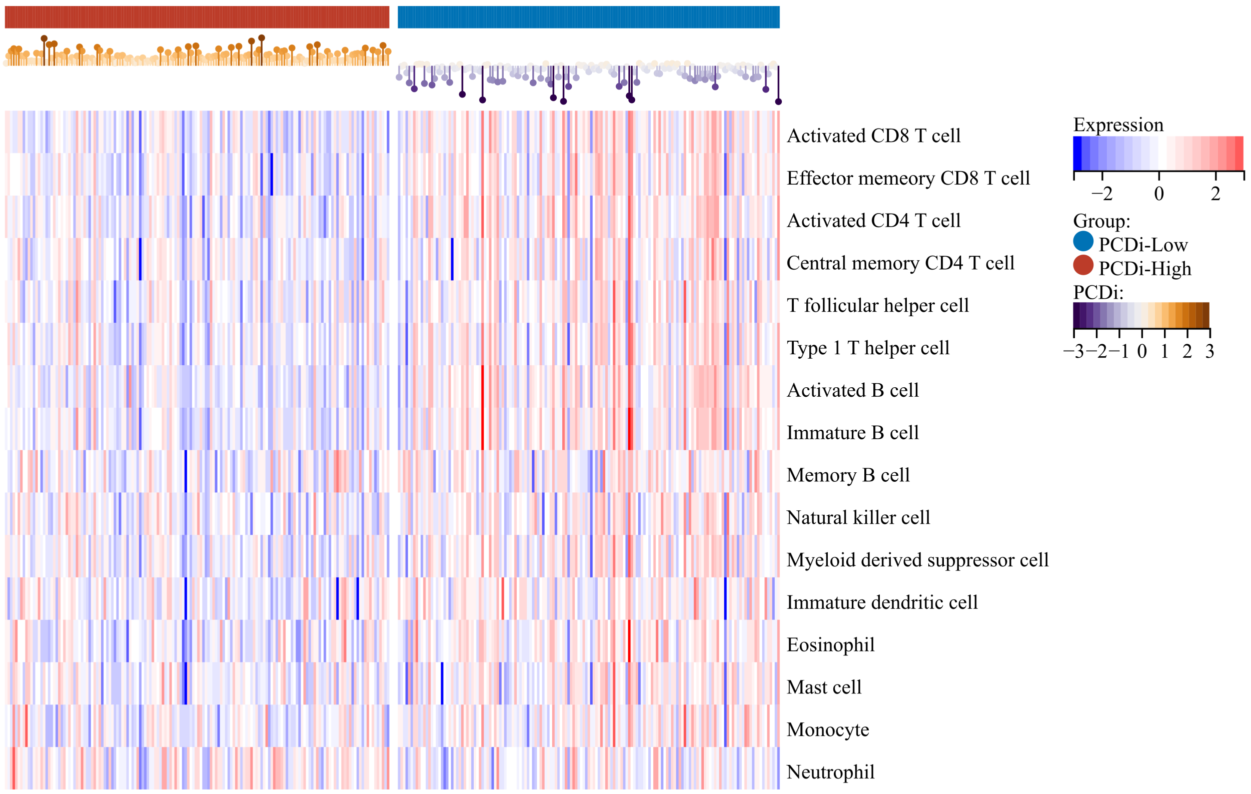


Figure S10. The expression profiles of different tumor immune infiltrates between PCDi-High and PCDi-Low groups through ssGSEA algorithm.

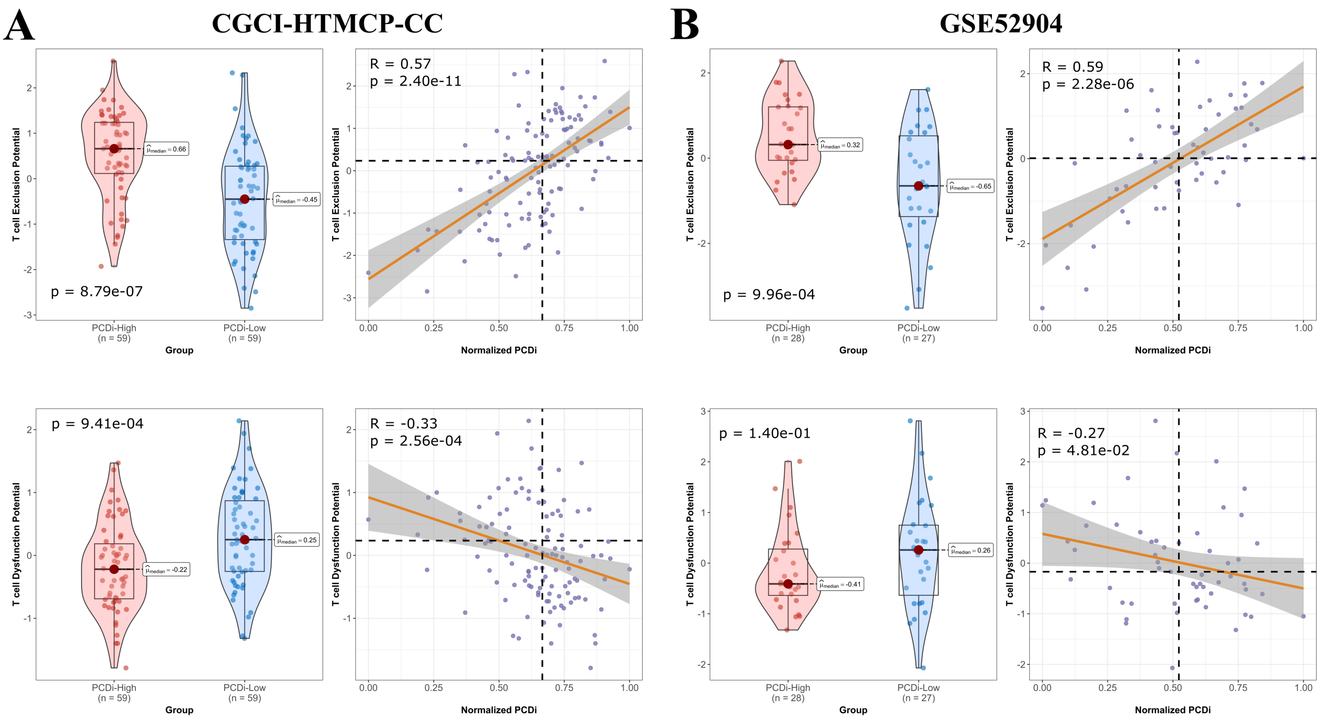


Figure S11. Violin and scatter plots displaying the association between PCDi and T cell dysfunction potential and exclusion potential. (A). CGCI-HTMCP-CC dataset. (B). GSE52904 dataset.

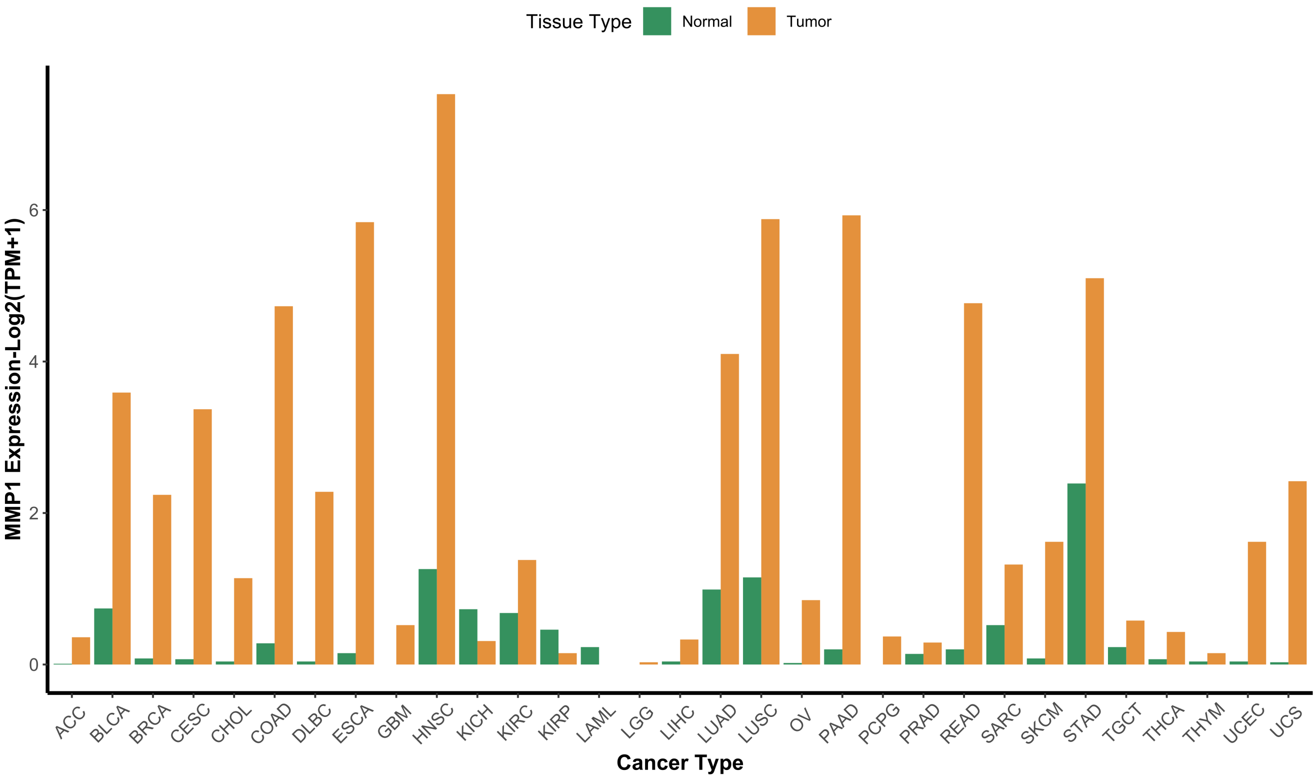


Figure S12. MMP1 expression levels in different cancer types. The expression data of MMP1 was obtained from GEPIA2 database.
